# To be climate-friendly, food-based dietary guidelines must include limits on total meat consumption – modeling from the case of France

**DOI:** 10.1186/s12966-025-01786-9

**Published:** 2025-07-09

**Authors:** Emmanuelle Kesse-Guyot, Julia Baudry, Justine Berlivet, Elie Perraud, Chantal Julia, Mathilde Touvier, Benjamin Allès, Denis Lairon, Serge Hercberg, Hélène Fouillet, Philippe Pointereau, François Mariotti

**Affiliations:** 1 INSERM, INRAE, CNAM, Center of Research in Epidemiology and StatisticS (CRESS), Nutritional Epidemiology Research Team (EREN), Université Sorbonne Paris Nord and Université Paris Cité, Bobigny, 93017 France; 2 Public Health Department, Avicenne Hospital (Assistance Publique- Hôpitaux de Paris (AP-HP)), Bobigny, France; 3 Aix Marseille Université, Inserm, INRAE, C2VN, Marseille, 13005 France; 4 Université Paris-Saclay, AgroParisTech, INRAE, UMR PNCA, Palaiseau, 91120 France; 5 Solagro, 75, Voie TOEC, CS 27608, F-31076, Toulouse Cedex 3, France

**Keywords:** Diet, Optimization, Mitigation, Greenhouse gas emissions, Dietary guidelines, Healthy diet

## Abstract

**Background:**

Although food-based dietary guidelines (FBDG) include guidelines for meat consumption, they most often do not explicitly include environmental considerations. For instance, in France, FBDG recommend consuming no more than 500 g of red meat and 150 g of processed meat per week. This study uses modeling to investigate the range of greenhouse gas emissions (GHGe) that can be achieved under FBDG compliance.

**Methods:**

The study analyzed data collected in 2014 from 29,413 NutriNet-Santé participants to assess their adherence to the French FBDG. GHGe, cumulative energy demand (CED), and land occupation (LO) for organic and conventional foods were obtained from the DIALECTE database. First, diets adequate in nutrients, culturally acceptable, and consistent with FBDG were modeled while minimizing or maximizing GHGe. Then, the spectrum of diets between minimum and maximum GHGe was explored while minimizing total departure from the observed diet with a gradual constraint on GHGE using the same other constraints. Environmental, economic (monetary cost), nutritional, and health criteria (Health risk score denoting long-term risk for health associated with diet) were then estimated for each diet.

**Results:**

The average observed adequacy to FBDG was low (19%, SD = 25%) and GHGe were 4.34 (SD = 2.7%) kgCO2eq/d. Under nutritional, acceptability and FBDG constraints, the GHGe range of the diets varied from 1.16 to 6.99 kgCO2eq/d, depending up to ∼ 85% on the level of meat consumption. A similar shape was observed for CED, LO, and Health Risk Score, but costs were consistently higher than in the observed diet, and exhibited a U-shape. A greater proportion of organic foods was noted in the lower-emission diet; however, this proportion was low in the meat-rich, high-emission diet. At isoenergetic diets, the diet with the lowest emissions had more vegetables, whole grains, and plant-based substitutes.

**Conclusions:**

While French dietary guidelines contribute, on average, to mitigating climate change and promoting health, this study emphasizes levers in recommended food consumption to more efficiently reduce diets’ GHGe and points to total meat as the critical issue to better account for pressure on climate change. Other environmental pressures should also be taken into account when designing dietary guidelines.

**Supplementary Information:**

The online version contains supplementary material available at 10.1186/s12966-025-01786-9.

## Highlights

The average greenhouse gas emissions of the observed diets was 4.34 (SD = 2.70) kgCO2eq/d, with an energy intake of 2080 Kcal/d.

The diet that closely resembled the observed diet under the dietary guidelines, nutrient and acceptability constraints had emissions of 5.15 kgCO2eq/d.

Modeled diets that complied with dietary guidelines and nutrient and acceptability constraints had emissions ranging from 1.16 kgCO2eq/d to 6.99 kgCO2eq/d.

All modeled diets had higher consumption levels of fruit, vegetable oils, pulses, and wholegrain products.

The diets minimizing and maximizing greenhouse gas emissions, and the range of modeled diets in between, differed in their level of beef/lamb, refined cereals, fruit, pork, and snack products.

The level of meat, especially beef/lamb, explained most of the difference (up to ≈ 85%) in greenhouse gas emissions between the models.

The level of total meat consumption decreased progressively across models that imposed a decrease in greenhouse gas emissions.

**Supplementary Information:**

The online version contains supplementary material available at 10.1186/s12966-025-01786-9.

## Introduction

The food systems are crippling the environment, pushing us beyond critical planetary boundaries and accelerating environmental decline [1, 2]. Food production is a major driver, and six out of nine planetary boundaries have already been breached [3–5]. For instance, food systems account for 34% of greenhouse gas emissions (GHGe) [6] and 70% of blue water usage [7]. Additionally, land overuse and reliance on synthetic inputs (fertilizers and pesticides) are driving biodiversity loss at an alarming rate [8].

Beyond environmental concerns, diet significantly contributes to the burden of disease [9, 10].

In that context, many countries have developed dietary guidelines in recent decades to guide populations towards healthier diets [11]. However, these guidelines frequently fail to fully account for the profound influence of agriculture and dietary patterns on the environment, despite the intricate interplay between these factors [11]. Few dietary guidelines were designed while accounting for the aim of minimizing environmental impact when setting consumption targets [12, 13].

Although there is a wealth of research on the dietary environmental burden associated with adherence to dietary guidelines, environmental impact estimates vary depending on methodological factors. It remains unclear to what extent following dietary guidelines aligns with reducing environmental pressures. Recently, Springmann et al. reported that compliance with most of these official dietary guidelines would yield only a modest 13% reduction in greenhouse gas emissions on average (geographical range: −34% to + 35%), as compared to the current situation [14].

In France, the French High Council of Public Health (HCSP, Haut Conseil de la Santé Publique) updated national dietary guidelines in 2017 [15]. The consumption limits recommended by the HCSP were based on scientific literature about the relationships between diet and long-term health, as well as healthy eating patterns as modelled by the French Food Safety Agency (ANSES) [16]. This modeling aimed to optimize diets by considering various factors, such as meeting nutrient reference intakes, establishing relationships between food group consumption and long-term health, limiting exposure to specific contaminants, and evaluating acceptable consumption levels. Although French dietary guidelines did not explicitly consider environmental pressures when they were implemented, we previously showed that diets closely following these guidelines had an overall reduced environmental footprint compared to non-compliant diets (comparing high versus low adherents: -46% GHGe) [17]. However, because this result was based on observed data, it does not mean that adherence to dietary guidelines necessarily implies low-emission diets.

The assessment of the alignment of these principles with existing food-based dietary guidelines (FBDG) has recently been investigated [11, 18, 19]. For instance, the FAO and WHO have established a list of 16 principles for sustainable healthy diets [20] covering health (8 items), environmental (5 items), and sociocultural (3 items) aspects. A recent report on the dietary guidelines of 83 countries found that no country addressed all 16 of these guiding principles in its documents, and that the FBDG of some countries, such as France, did not fully align with the FAO principles [11]. Only 45% of FBDG documents mentioned environmental preservation, and the vast majority lacked consistency with sustainability.

This study aimed to explore the range of GHGe resulting from diets that follow the FBDG using optimization modeling. By analyzing diets that meet both nutrient and acceptability constraints alongside all individual FBDG recommendations, we identified the minimum and maximum levels of GHGe. We then investigated which diet characteristics were linked to the gradual changes in GHGe.

## Materiel & method

### Population and ethics approval declaration

This study was conducted on a sample of adults from the web-based prospective nutritional NutriNet-Santé cohort [21]. The study began in 2009 and recruitment is still open. The participants are volunteers recruited from the general French population. This study is conducted in accordance with the Declaration of Helsinki, and all procedures were approved by the Institutional Review Board of the French Institute for Health and Medical Research (IRB Inserm 0000388FWA00005831) and the National Commission on Informatics and Liberty (Commission Nationale de l’Informatique et des Libertés, CNIL 908450 and 909216). Electronic informed consent was obtained from all participants. The NutriNet-Santé study is registered in ClinicalTrials.gov (NCT03335644).

Sociodemographic characteristics, including age, education (< high school diploma, high school diploma, and post-secondary graduate), lifestyles, i.e. smoking status (former, current, or never-smoker) and physical activity level assessed using the International Physical Activity Questionnaire [22] as well as anthropometrics data [23], are collected using pre-validated questionnaires each year [24, 25]. The participants were asked to report their total monthly income from different sources, such as salary, rental income, family allowance, or social benefits. To determine the monthly household income, the household unit was defined according to the National Institute of Statistics and Economic Studies (INSEE) guidelines [26]. The first adult in the household was allocated one household unit, while other individuals aged 14 years or older were allocated 0.5 units, and children below 14 years were allocated 0.3 units. We reported data closest to the FFQ (Food Frequency Questionnaire, see below).

### Dietary data

The dietary data were collected in 2014 via a self-administered semi-quantitative FFQ, aiming to distinguish organic (under the official label) and conventional food consumption [27]. This tool is based on a 264-item food frequency questionnaire, previously tested against repeated 24-hour dietary records (DRs), and showed acceptable reproducibility and relative validity [28]. Participants reported how often they consumed the standard portion size recommended. This frequency pertained to their typical eating habits over the last year, measured on a scale that included yearly, monthly, weekly, or daily categories as applicable. They were instructed to provide just one response. For quantity, participants were also helped by validated photographs showing different portion sizes [27]. The FFQ used was improved by a five-point scale to evaluate the food production mode [27]. For each food item, participants reported the frequency of food consumed (over the past 12 months) as organic by ticking the following modalities: “never”, “rarely”, “half-of-time”, “often” or “always” in response to the question ‘How often was the product of organic origin?’. Weight was allocated to each frequency modality, i.e., 0, 25, 50, 75, and 100%, respectively. The nutritional composition of each item was determined by combining the published NutriNet-Santé food composition table (> 3500 items) (Etude Nutrinet-Santé) with the FFQ-items as the weighted mean of the nutritional content of all corresponding foods. Weights were the frequencies of consumption in the overall NutriNet-Santé population.

Under- and over-reporters were defined as participants with a ratio between energy intake and energy requirement below or above cut-offs previously identified (0.35 and 1.93) corresponding to the 1st and 99th percentile of the ratio distribution [27].

### French food-based dietary guidelines and PNNS-GS2

In France, the High Council of Public Health published the revised version of the dietary guidelines for adults in 2017 [15], including both specific food consumption targets and general guidelines such as: *“to promote dietary sustainability in the dietary guidelines: opt for raw (unprocessed)*, *seasonal food products*, *rely on short supply chains and low-input production methods*, *i.e. with a restriction in inputs”.*

To reflect the level of adherence to these dietary guidelines, a validated FBDG adherence score (sPNNS-GS2 ) has been previously developed and validated [29], and showed strong association with a wide range of health outcomes [29–31].

The sPNNS-GS2 (theoretical range: -∞ to 14.25) consists of 6 adequacy components and 7 moderation components. The components are weighted according to the level of epidemiological evidence for the association with health, and a penalty for energy intake is also given. if it exceeds nutritional needs. It includes components related to fruits and vegetables, pulses, whole grains, nuts, fish, red meat, processed meat, sweet products, sweet drinks, added lipids, alcohol, dairy products, and salt. Scoring and computation have been extensively described elsewhere [29] and are presented in Supplemental Tables 1 and Supplemental Method 1.

For easier reading, the PNNS-GS2 will be called the FBDG adherence score.

### Environmental pressure data

Environmental indicators assessment related to food production was computed using life cycle analysis (LCA) using the DIALECTE database developed by Solagro [32]. GHGe (kg of CO_2_ equivalents (CO_2_eq)), cumulative energy demand (MJ), and land occupation (m^2^) for organic and conventional food production were calculated. Only the production stages have been considered due to a lack of data regarding food production methods for other steps. The packaging, transport, treatment, storage and recycling stages were not included in the scope of the LCA. Extensive details and raw data have been provided elsewhere [33] and are provided in Supplemental Method 2.

### Food prices

A database containing the price of each food item was created. The database considers where the food was purchased and the farming method used (organic or conventional). It is based on the Kantar Worldpanel^®^ purchase database, which includes information from 20,000 households. The expenditures from Kantar were used to derive prices for each of the 264 items, in organic and conventional, according to purchase locations (superstores, supermarkets, and specialized stores). Furthermore, additional prices were gathered by volunteers from the Bioconsom’acteurs association concerning food groups supplied through short channels (e.g., local markets or associations supporting small farming) [34].

### Diet modeling

The optimized diets were identified using the procedure SAS/OR ^®^*optmodel* (version 9.4; SAS Institute, Inc.). A non-linear optimization algorithm with multistart was used to select a solution that is not only a local minimum. The solutions of the optimization procedure provided the consumption in 47 food groups and the % of organic for each of these groups (as the GHGe for a given food group varies depending on the production method). The models’ input parameters were the mean and 95th percentile of the weighted (see below) observed consumption, and the nutrient content of the 47 groups (calculated by weighting the nutritional values of the items constituent of the group by the population consumption of each item). Each group’s GHGe (organic or conventional) was calculated in the same way.

#### Optimization process and objectives

- First, we identified the modeled diet as closest to the observed diet while complying with all the nutritional, acceptability, and FBDG constraints, intended to explore what this diet would be like without any limitations regarding GHGe. This model minimized the total departure (TD) from the observed diet under all these constraints (TD model), according to the formula (1):
  1 $$Min\,TD = {\sum\nolimits_i^{47} {\left[ {\frac{{Op{t_i} - Od{s_i}}}{{S{D_i}}}} \right]} ^2}$$
  Where $\:{Opt}_{i}$ and $\:{Obs}_{i}$ respectively denote the optimized and observed daily consumptions of the food group *i*, with SD_*i*_ being its standard deviation in the observed situation.
- Second, we identified the optimized diets with the lowest and highest GHGe values while complying with all the nutritional, acceptability, and FBDG constraints, by minimizing or maximizing GHGe under all these constraints, respectively (MinGHGe and MaxGHGe models).
- Finally, we identified a full spectrum of modeled diets that vary in greenhouse gas emissions, spanning from the previously determined minimum to maximum values (from 1.2 to 6.8 kgCO_2_eq/d), and complying with all the nutritional, acceptability, and FBDG constraints, by minimizing the total departure (TD) from the observed diet under all these constraints and an additional constraint on GHGe to cover its whole possible range, using a grid search by 0.2 kgCO_2_eq/d. GHGe increments.

#### Models constraints

- Nutrient constraints
  The nutrient constraints, which included daily intakes of energy and a set of nutrients, were based on the lower and/or upper ANSES 2016 dietary reference intakes [35]. A specific constraint imposed energy intake to be between + 8% and − 8% of energy requirements. Lower bounds were defined as either recommended dietary allowance (population reference intake), adequate intake, or lower bound of reference range for the intake in the French population [35]. Upper bounds were defined as the maximum tolerable intakes for vitamins and minerals or the upper limit of the reference intake range. For zinc and iron, bioavailability was considered using validated equations (Supplemental Method 3 and Supplemental Method 4) [36, 37]. A minor acceptable lowest limit than nutritional references based on deficiency intake has been defined for bioavailable zinc and iron as previously published [38]. The lower threshold values used in this context pertain to a deficiency prevalence of < 5%. This approach offers greater flexibility in identifying diets that are considered healthier overall despite the higher prevalence of iron deficiency anemia [38]. All reference values used are shown in Supplemental Table 2.
  Of note, the French nutritional reference values for adults are based on the specific physiological requirements of males and females, and established separately for each gender [35]. Additionally, the reference values are further differentiated for females based on their iron requirements, with a distinction made between females with high and low/moderate iron requirements. To create new nutritional reference values more representative of the average individual, we have derived a weighted average of requirements for males, females with high iron requirements, and females with low/moderate iron requirements. Therefore, the reference values for each nutrient for this average individual are defined as the weighted average requirements of males and females (Supplemental Table 2). For adequate intake, based on observed mean intake, the lower limit was set at the 5th weighted percentile value of the overall population.
- FBDG constraints
  To comply with official French dietary guidelines [15], models were additionally constrained on the consumption of different food groups using thresholds set by the official French FBDG, quantitatively translated for the FBDG adherence score computation (see Supplemental Table 1).
  - Consumption of fruit and vegetables (including 100% fruit juice up to a maximum of one portion) ≥ 400 g/d.
  - Consumption of 100%fruit juice ≤ 150 g/d.
  - Consumption of nuts ≥ 30 g/d.
  - Consumption of pulses ≥ 400 g/wk (i.e. ≥57 g/d).
  - Consumption of dairy products: 2 portions/d (with a portion for milk = 150mL, cheese = 30 g, yogurt = 125 g, “*Petits Suisses*”=120 g, cottage cheese = 100 g).
  - Consumption of wholegrain products ≥400 g/wk (i.e. ≥57 g/d).
  - Consumption of red meat ≤ 500 g/wk (i.e. ≤71.4 g/d).
  - Consumption of processed meat ≤ 150 g/wk (i.e. ≤21.4 g/d).
  - Consumption of total seafood 2 portions/wk (with a portion = 100 g, i.e. 28.57 g/d).
  - Consumption of fatty fish 2 portions/wk (with a portion = 100 g, i.e. 14.28 g/d).
  - Consumption of added fat (added lipids) ≤16% of total energy intake.
  - Consumption of sweet drinks (including other fruit juice, sweet and artificially sweetened beverages) = 0.
  Of note, no constraints were imposed on salt and sugary foods, as the nutrient-related constraints already considered the limitations of sodium and sugar.
- Acceptability constraints
  In our study, the acceptability constraints are not very severe compared with those used by other authors [39–41] and instead reflect the feasibility in the population. Thus, to prevent the models from giving aberrant values (i.e., excessive intakes for some food groups), the maximum possible intake for each food group was set at the 99th percentile of observed consumption distribution. For cereals (refined and wholegrain), a so-called “coupling” limit allowed inter-group substitution so that each can exceed its 99th percentile while only the sum was constrained to the 99th percentile. In addition, as pulse consumption is very low in the observed diet, no acceptability constraint was used for this food group.
- Sensitivity analyses
  Two sensitivity analyses were conducted. First, a sensitivity analysis was also performed to compare the results under different FBDG constraints to identify the changes in the maximum GHGe values when further restricting the total meat consumption, from 500 (main scenario) to 400, 300, or 200 g/wk. Then, the full spectrum of healthy modeled diets of increasing imposed GHGe values complying with nutritional, acceptability, and FBDG constraints, by minimizing the total departure (TD), was reanalyzed with the use of the 95th percentile of the food group consumption distribution as acceptability criteria.

### Descriptive statistics

The observed situation was based on the data of participants in the NutriNet-Santé who had completed the FFQ between June and December 2014 (*N* = 37,685), with no missing covariates (*N* = 37,305), who were not under or over-energy reporters (*N* = 35,196), living in mainland France as environmental indicators were estimated for mainland France (*N* = 34,453), and with information as regards the individual place of purchase of food groups allowing the computation of the dietary monetary cost (*N* = 29,413). A flowchart is provided on Supplemental Fig. 1. Observed sociodemographic and lifestyle characteristics of the sample were estimated as mean (SD) or percentage according to sex-specific quintiles of the FBDG adherence score.

The modeled diets were described in terms of food group consumption (the 47 food groups used for optimization were grouped into 25 groups for clarity purposes), nutrient intakes, potential health risk, assessed using the Health Risk Score (HRS), compared to the theoretical maximal risk exposure level (TMREL) of the 2019 Global Burden of Diseases (GBD) study, environmental pressures (GHGe, CED, and LO) as well as monetary cost of the diets. The HRS is presented in Supplemental Method 5.

All statistical analyses were performed using SAS^®^ (version 9.4; SAS Institute, Inc., Cary, NC, USA) and Figures were developed using R version 3.6.

## Results

### Observed diets

In the observed situation, the weighted mean (SD) age was 55 years (14), and FBDG adherence score was 2.28 (3.57). The average GHGe was 4.34 ± 2.70 kgCO2eq/d (at the farm perimeter) (Table 1). The sample characteristics by FBDG adherence score quintiles are presented in Supplemental Table 3. Better adherence to dietary guidelines was associated with older age and higher levels of education, income, and physical activity. As regards smoking and living with a partner, a negative association was observed. Adherence was negatively associated with daily energy intake, but positively associated with consumption of organic foods and the proportion of plant protein in total protein intake.

Participants in the Q5 had higher or much higher consumption of plant products, especially fruits and vegetables, oilseeds, pulses, whole grains, and plant substitutes, compared with individuals in Q1. Higher adherence was associated with higher GHGe, even after adjusting for energy intake.

**Table 1:** Characteristics of observed diets and main modeled diets

	Obs	min TD^1^	min GHGe^2^	max GHGe^3^
GHGe (kgCO_2_eq/d)	4.34 (2.70)	5.15	1.16	6.99
GHGe (kgCO_2_eq/d)/1000kcal^4^	2.09	2.17 (+ 4%)	0.49 (-77%)	2.82 (+ 35%)
Land occupation (m²/d)	11.36 (7.35)	12.93	4.43	20.09
Cumulative energy demand (MJ/d)	18.45 (7.98)	25.14	10.61	33.52
Energy intake (Kcal/d)^5^	2080 (661)	2373	2373	2482
% organic food in the diet	28 (27)	0	76	24
HRS^6^	0.75 (0.30)	0.39	0.09	0.38
Monetary cost of the diet (€/d)	7.99 (3.07)	8.90	11.72	13.5
Plant protein (% of total protein)	33 (14)	56	82	43
Consumption (g/d)				
Alcoholic beverages	128 (180)	1	1	1
Animal fat	6 (7)	0	0	0
Beef	44 (43)	69	0	71
Refined cereals	140 (99)	254	266	325
Dairy products	185 (139)	96	67	63
Eggs	11 (12)	3	0	0
Fish	48 (46)	29	29	29
Fruit	283 (252)	446	369	666
Fruit juice	85 (118)	101	150	150
Milk	59 (135)	0	0	0
Nuts	8 (16)	15	15	15
Offal	2 (7)	2	0	0
Mixed dishes^7^	29 (36)	0	0	0
Other fat	7 (9)	0	0	0
Pork	51 (4)	0	0	14
Potatoes	24 (25)	0	0	0
Poultry	24 (26)	26	0	106
Pulses	17 (32)	57	143	57
SFF^8^	73 (58)	66	42	0
Snack	11 (16)	0	0	49
Sweet drinks^9^	47 (111)	0	0	0
Substitutes	40 (138)	3	157	5
Vegetable fat	23 (16)	46	50	49
Vegetables	355 (236)	930	930	930
Wholegrain products	58 (75)	191	255	196

Abbreviations: GHGe, greenhouse gas emissions; HRS, Health Risk Score; Obs, observed diet (mean, SD); SFF, Sweet and fat foods, SFF, Sweet and fat foods

^1^Min TD is the model under nutritional, dietary guidelines and acceptability constraints minimizing the total departure from the observed diet

^2^Min GHGe is the model under nutritional, dietary guidelines and acceptability constraints minimizing the GHGe

^3^Max GHGe is the model under nutritional, dietary guidelines and acceptability constraints maximizing the GHGe

^4^ Values in parentheses are relative difference to the observed situation

^5^ The comparison between the modeled energy intake and the observed value must be approached with caution, given that the observed value is derived from a validated Food Frequency Questionnaire (FFQ), which is inherently subject to measurement error

^6^HRS (%) is the normalized distance to the theoretical minimum-risk exposure levels (TMREL) from the Global Burden of Diseases, expressed in % (i.e., HRS = 0% when the diet is at minimal risk by meeting all the TMREL and HRS = 100% when the diet is at maximal risk by deviating from them at most)

^7^Mixed dishes include sandwiches, dishes such as pizza, hamburger, ravioli, panini, salted pancake

^8^Sweet and fat foods (SFF) include croissants, pastries, chocolate, biscuits, milky desserts, ice cream, honey and marmalade, cakes, chips, salted oilseeds, salted biscuits

^9^Sweet drinks include fruit nectar, syrup, soda (with or without sugar)

### Modeled diets

When modeling a diet (model TD, i.e. as closely as possible to the observed diet), under nutritional constraints and PNNS recommendations, emissions increased to 5.15 kgCO_2_eq/d i.e. +4%/1000 kcal compared to the observed diet (Table 1). When minimizing and maximizing GHGe, diets that complied with nutritional, acceptability constraints, and dietary recommendations, had emissions ranging from 1.16 kgCO_2_eq/d (model MinGHGe) to 6.99 kgCO_2_eq/d (model MaxGHGe) (Table 1), i.e. -76.7 to + 34.8%/1000 kcal compared to the observed diet.

Similar results were observed for LO and CED. The TD model contained no organic food (as by construction, no constraints depending on the mode of production were introduced to the model), while from MinGHGE to MaxGHGE models, %organic food products varied from 24% (MaxGHGE) to 76% (MinGHGE).

In the TD model (Table 1), certain food items such as alcoholic beverages, animal fats, milk, other fats, pork, potatoes, snack foods, and soft drinks were excluded compared to the observed diet due to the nutritional and PNNS recommendations constraints. Whereas the red meat is < 500 g/wk by the PNNS recommendations, the total meat intake (beef/lamb, poultry) in the TD model was high, 97 g/d (i.e., ≈ 680 g/wk), due to poultry.

The excluded foods were similar in both the MinGHGe and MaxGHGe models. For all three models (i.e., minimizing total departure – TD–, minimizing GHGe – MinGHGe–, and maximizing GHGe – MaxGHGe), there was a systematic increase, compared to the observed diet, of the consumption of fruit, fruit juices, vegetable oil, pulses, and wholegrain products. Conversely, consumptions of eggs, fish, dairy products, and fatty and sweet products were reduced. The MinGHGe and MaxGHGe models differed in their level of beef/lamb, refined cereals, fruit, pork, and snack products, for which we saw an increase in consumption in the MaxGHGe model. On the contrary, pulses, wholegrain products, and plant-based substitutes (especially soya-based products) experienced a decrease. Notably, there was a shift towards plant-based diets from the MaxGHGe to MinGHGe models, as expressed by the higher % of protein derived from plant sources from 43 to 82%.

**Fig. 1:**
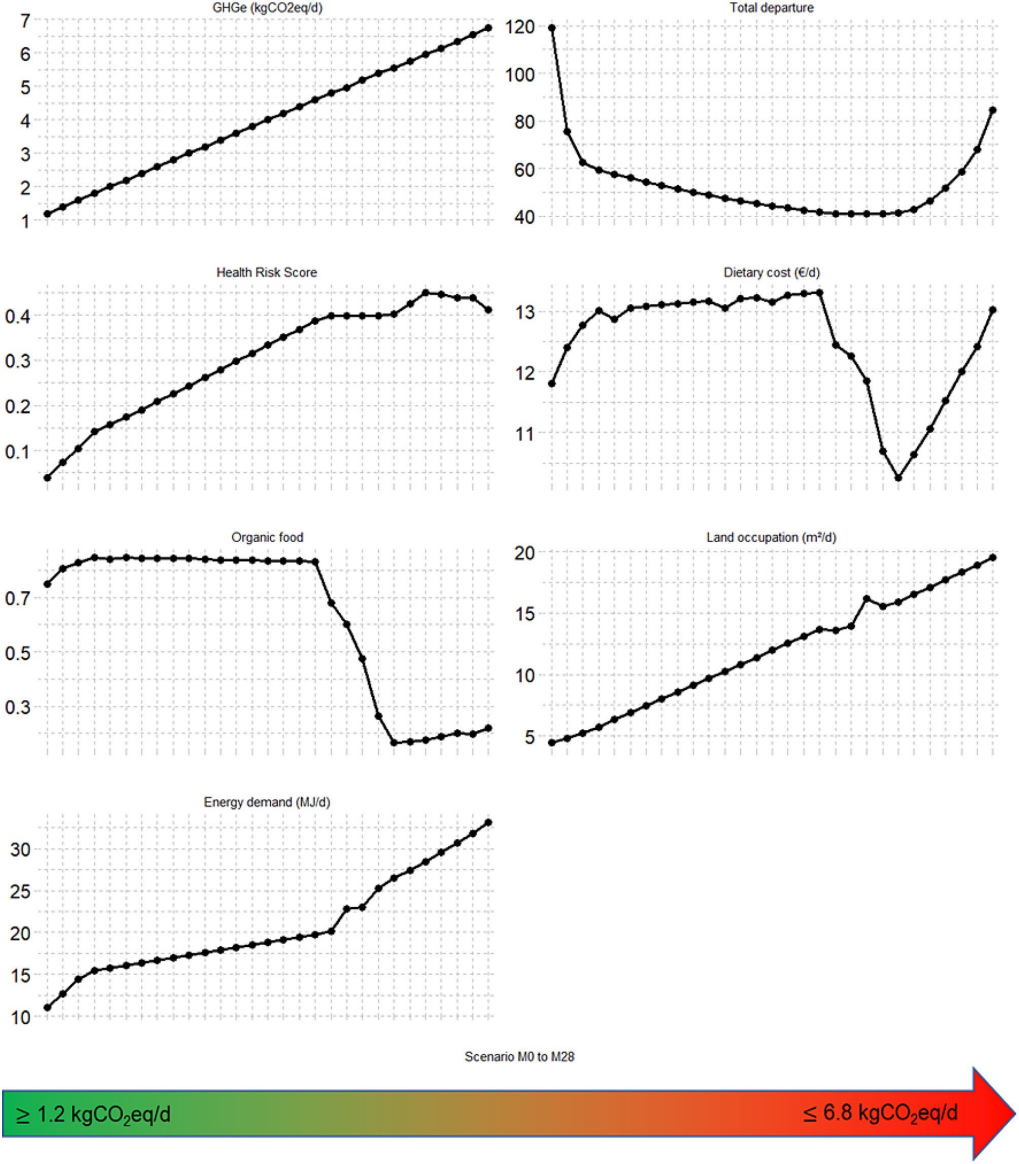
Characteristics of modeled diets adhering to dietary guidelines at different levels of GHGe^1–2^ Abbreviations and units: CED, cumulative energy demand (MJ/d); GHGe, greenhouse gas emissions (kg CO2 eq/d); HRS, health risk score (a lower value is healthier); LO, land occupation (m^2^/d); Organic Food, proportion of organic food in the diet; TD, total departure of observed diet. Cost is in euros/d, SFF, Sweet and fat foods ^1^M0 to M28 denote models imposing GHGe of 1.2 to 6.8 kgCO2eq/d by increments of 0.2 ^2^HRS (%) is the normalized distance to the theoretical minimum-risk exposure levels from the Global Burden of Diseases, expressed in % (i.e., HRS = 0% when the diet is at minimal risk because meeting all the TMREL and HRS = 100% when the diet is at maximal risk by deviating from them at most)

Then, the spectrum of diets with GHGe from minimum to maximum was examined by applying a gradual constraint on GHGe between the two limits. Figure 1 describes various indicators for the GHGe-imposed diets. Specifically, higher GHGe correlated with increases in other environmental indicators such as LO and CED. Similarly, their HRS (diet associated with risk to health) increased with GHGe. Conversely, the proportion of organic food in the diet increased non-linearly and then fell drastically. Additionally, the distance from the observed diet exhibited a U-shaped curve, with the levels furthest from the observed diet found at low and high GHGe extremes. The monetary cost of the diet did not correlate linearly with greenhouse gas emissions; rather, it entailed high expenses for diets set at either very low or very high emissions, while costs were lower for diets between 4.8 and 6 kgCO_2_eq/d.

**Fig. 2:**
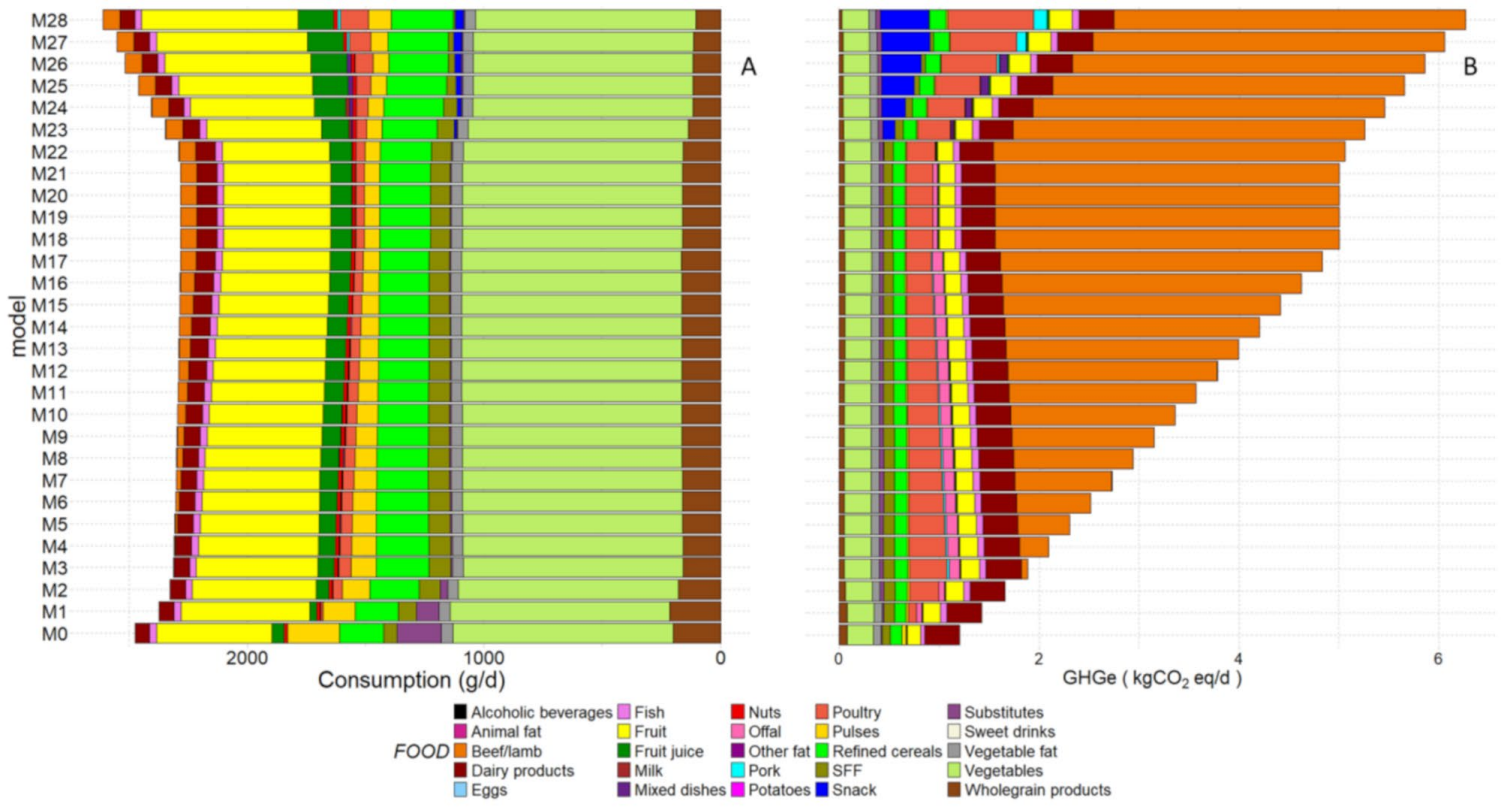
Food group consumptions and contribution to GHGe in modeled diets adhering to dietary guidelines of graded GHGE values ^1,2^ Panel A represents food consumption (g/d) in each modeled diet and Panel B represents the corresponding contribution to GHGe Abbreviations: GHGe, greenhouse gas emissions; M: model; SFF, Sweet and fat foods The 47 food groups are pooled into 25 broader food categories for clarity ^1^Mixed dishes include sandwiches, dishes such as pizza, hamburger, ravioli, panini, salted pancake, sweet and fat foods (SFF) including croissants, pastries, chocolate, biscuits, milky desserts, ice cream, honey and marmalade, cakes, chips, salted oilseeds, salted biscuits, and sweet drinks include fruit nectar, syrup, soda (with or without sugar) ^2^M0 to M28 denote models imposing GHGe of 1.2 to 6.8 kgCO2eq/d by increments of 0.2

The food group composition of the modeled diets with gradually imposed GHGe is shown in Fig. 2 (Panel A) and Supplemental Table 4. The Fig. 2 (Panel B) details the contribution of food groups to total GHGe for the GHGe-imposed diets.

A gradual increase in GHGe was linked to progressive variations in most types of consumption. In the models with the lowest emissions, meat of all types is minimally present, and emissions arise from dairy products, milk, and then poultry when it appears. As emissions rise, plant consumption and its contribution to GHGs remain similar or increase marginally. Notably, the increase in GHGe was associated with an increase in beef/lamb consumption, along with a reduction of fruit juices and poultry, while pulses and plant-based substitutes increased. In addition, a slight decrease in the consumption of wholegrain cereals and an increase in refined cereals were observed. Vegetable and fish consumption remained steady. Meanwhile, some food categories, including dairy products, offal, and sweet or fatty items, showed a bell-shaped distribution. In the models, vegetables, fish, and oilseeds were positioned at either the upper or lower limits. Certain foods, such as animal fats, eggs, and potatoes, were omitted from the modeled diets. Although pork consumption lacked a clear trend, it was most common in the diet with the highest emissions.

According to model M21 (≈ 5.4 kgCO2eq/d), the maximum limit for dairy products, processed meat, and red meat has been reached. As a result, poultry and poultry-based snack products are now the primary contributors to the increase in GHG emissions. The gradual increase in GHGe corresponded to a higher increase in the contribution of meat (beef/lamb, poultry, pork) and dairy products, from ∼ 30% (in M0) up to ∼ 85% (in M28). The gradual increase in GHGe corresponded to a higher increase in the contribution of meat (beef/lamb, poultry, pork) and dairy products, from ∼ 30% (in M0) up to ∼ 85% (in M28).

Supplemental Fig. 2 shows, for illustrative purposes, the contributions of food groups to nutrient intakes across different modeled diets.

In the sensitivity analyses, decreasing the upper limit for total meat consumption from 500 to 200 g/wk when identifying the healthy diets induced a decrease in their maximum total diet-related GHGe value, from 6.44 (M500) to 4.38 kgCO_2_eq/d (M200) Table 2). There were concomitant decreases in land occupation, energy demand, and HRS, while the percentage of plant protein and the percentage of organic food increased. The optimized diets were similar, except for a decrease in cereals, substitutes, and meat (regardless of type), and an increase in pulses and whole grains. To comply with nutritional references, beef/lamb was selected while poultry and pork were excluded. Additionally, lowering the maximum amount of food that could be consumed from the 99th to the 95th percentile in the acceptability constraints had only a slight impact on the results (Supplemental Table 5). The differences were minor, primarily affecting the diets with high GHGe. For example, the amount of vegetables decreased, and there was a shift towards more pulses, and the amount of poultry decreased, resulting in no solution beyond 5.6 kgCO2eq/d.

**Table 2:** Description of the diet models, maximizing GHGe constrained for different levels of total meat ^1^

	M500	M400	M300	M200
GHGe (kgCO2eq/d)	6.44	5.75	5.07	4.38
Land occupation (m²/d)	18.47	16.25	14.02	11.81
Cumulative energy demand (MJ/d)	29.15	27.81	26.44	25.06
Energy intake (kcal/d)	2433.69	2405.18	2375.69	2373.86
% organic food in the diet	24	24	24	27
HRS^2^	0.39	0.34	0.30	0.20
Plant protein (% of total protein)	53.27	54.96	56.80	60.16
Consumption (g/d)				
Alcoholic beverages	1	1	1	1
Animal fat	0	0	0	0
Beef	71	57	43	29
Cereals	306	295	282	247
Dairy products	102	102	102	102
Eggs	0	0	0	0
Fish	29	29	29	29
Fruit	637	637	637	634
Fruit juice	150	150	150	150
Milk	0	0	0	0
Nuts	15	15	15	15
Offal	0	0	0	0
Mixed dishes^3^	76	82	88	92
Other fat	0	0	0	0
Pork	0	0	0	0
Potatoes	0	0	0	0
Poultry	0	0	0	0
Pulses	57	57	57	99
SFF^4^	0	0	0	0
Snack	49	49	49	49
Sweet drinks^5^	0	0	0	0
Substitutes	41	37	33	28
Vegetable fat	45	44	44	44
Vegetables	930	930	930	930
Wholegrain products	215	226	239	274

Abbreviations: GHGe, greenhouse gas emissions; HRS, health risk score; SFF, Sweet and fat foods

^1^ All models maximized GHGe under nutritional, dietary guidelines, and acceptability constraints. M500 to M200 refers to a maximum of 500 to 200 g of meat per week

^2^HRS (%) is the normalized distance to the theoretical minimum-risk exposure levels (TMREL) from the Global Burden of Diseases, expressed in % (i.e., HRS = 0% when the diet is at minimal risk by meeting all the TMREL and HRS = 100% when the diet is at maximal risk by deviating from them at most)

^3^Mixed dishes include sandwiches, dishes such as pizza, hamburger, ravioli, panini, salted pancake

^4^Sweet and fatty foods (SFF) including croissants, pastries, chocolate, biscuits, milky desserts, ice cream, honey and marmalade, cakes, chips, salted oilseeds, salted biscuits

^5^Sweet drinks include fruit nectar, syrup, soda (with or without sugar)

## Discussion

### GHG of French FBDG as compared to others

In the present study, we observed that it was possible to obtain nutritionally adequate diets that adhered to all recommendations of the French FBDG, with associated GHGe ranging from 1.6 to 6.8 kgCO^2^eq/d. This extensive GHGe range can be explained by the fact that the French FBDG do not have a low specific target for total meat but only recommend upper limits for red and processed meats that are relatively high (e.g. 500 g/wk) compared to other FBDGs, especially in countries where a strong emphasis already exists to promote environmental sustainability alongside health. For instance, in the Netherlands, it is recommended that individuals limit their consumption of all types of meat (i.e., including poultry) to 500 g per week [12]. Additionally, various countries have introduced stricter dietary guidelines. For example, the 2023 Nordic nutrition guidelines suggest restricting red meat intake to 350 g per week [42] and also advise cutting back on poultry. This is reflected in Finland’s unpublished recommendations and Denmark’s guidelines [43], which both advocate for a total meat limit of 350 g per week. Estonia takes a more extreme approach, recommending only 100 g of red and processed meat weekly, and preferring poultry.

Although poultry meat production generates less GHG than ruminant meat, its emissions per kilogram are still significant and much higher than those of plant-based foods [3, 6, 44].

Our results align with the extensive literature indicating that consuming animal products, primarily meat, is associated with very high GHGe [41, 45, 46]. This is the case even for diets following FBDG. For example, a study conducted in the Netherlands, based on the recommendations before their update, found that adhering to dietary guidelines could reduce the environmental impact for males aged 31–50 by up to 13%, while it might increase it by up to 5% for women aged 19–30. Conversely, adopting a meat-free version of the same diet based on the Dutch guidelines could reduce the environmental impact by 28–46% [47]. Following the Dietary Guidelines for Americans for an omnivorous diet does not necessarily lead to lower greenhouse gas emissions (GHGe), primarily due to the high levels of total meat, in stark contrast to the vegetarian version of the Dietary Guidelines for Americans [48].

Our results, along with others, underscore that following the French FBDG can lead to an extensive range of environmental pressures. For this reason, some countries have directly considered the environmental criteria, particularly GHGe, when developing their dietary guidelines, as part of diet modeling [11], unlike France.

For instance, the Netherlands has recently based its guidelines on optimization models that set maximum consumption levels for foods that produce high levels of greenhouse gases [12]. The United States has developed guidelines for broad food groups, such as the Protein Foods Group, which includes lean meat and poultry, eggs, seafood, beans, peas, lentils, nuts, seeds, and soy-based products. This has resulted in significantly different environmental footprints for the set of diets that comply with the guidelines for the “Protein Foods Group,” depending on the type of food within that group [49].

### Levers of the FBDG on GHG and healthiness

Here, we found that complying with FBDG while departing as little as possible from the usual diet led to a ∼ 4% increase in GHGe per 1000 Kcal compared to the observed diet. Thus, individuals wishing to enhance their adherence to FBDG with minimal adjustments to the current French diet may slightly increase climate pressure. This result aligns with the extensive scientific literature indicating that not all healthy diets are necessarily low-emission diets [14, 50, 51] and that there are significant variations in GHGe across FBDGs [14, 52].

When GHGe was also constrained, results indicated that plant-based diets resulted in lower emissions compared to those with substantial or minimal amounts of animal products, particularly ruminant meat, aligning with the scientific literature [41, 45, 46]. This is also consistent with recent work focusing on protein, which shows that a healthy diet (in terms of both nutritional adequacy and long-term health) that is richer in plant protein leads to lower environmental pressures [53]. In addition, our long-term health indicator (reflecting adherence to the 2019 Global Burden of Diseases’s TMRELs) showed that, within the limits of the FBDGs, a more plant-based diet, rich in fruits and vegetables, pulses, and whole grains, was associated with a lower health risk. This aligns with the literature documenting the health value of more plant-based diets [44, 54, 55]. It also highlights the fact that diets following dietary recommendations exhibit a wide range of health risks.

### Other issues remaining unresolved and implications

GHGe is generally seen as a strong indicator of global environmental pressures [56]. However, the climate mitigation approach should not overlook other equally essential indicators for achieving sustainable food systems, particularly water use, biodiversity conservation, and fisheries resources. Indeed, we recently demonstrated in an analysis of the trade-offs between reducing water use and reducing GHGe that discrepancies exist between modeled diets, depending on whether the modeling is guided by one parameter or the other [57]. Indeed, plant-based diets are generally better for both health and the environment; however, there are still potential conflicts regarding specific environmental criteria, particularly regarding water use [58]. Then, diets rich in plant-based foods may increase exposure to certain chemicals [59]. Additionally, other factors such as pollutants could also be included in models to limit health risks [16]. Finally, modeled healthy low-GHGe diets, characterized by a preference for organic foods over conventional options due to their lower GHGe, are rich in plant foods, as previously documented [60]. Thus, in the context of climate mitigation, it is important to consider dietary patterns but also production methods and potential improvements in agricultural practices. Furthermore, optimized diets that prioritize lower emissions and greater levels of plant products as organic, often come at a higher cost [34]. Even though it would reduce their exposure to synthetic pesticides, this raises concerns about affordability for consumers.

Moreover, compliance with dietary guidelines differs by food group. A 2016 study revealed that restricting red meat was the most frequently followed recommendation among French adults [61]. Conversely, guidelines concerning pulses, wholegrain products, and processed meats were largely overlooked. Individuals need better education about the environmental impact of their food consumption, as well as the significance of dietary recommendations and associated risks. The implementation of the recommendations must be based on a comprehensive set of public policies, and in the design of sustainable dietary guidelines, the focus should reside on a set of common objectives rather than separate ex-post assessments; this can be achieved using optimization methods similar to those employed for the 2019 FBDG for French adults [16].

### Assessment of FBDG in relation to the FAO principles

Beyond addressing environmental impacts, the FAO principles establish a list of targets to promote food sustainability [20]. In this context, several studies have recently evaluated the sustainability of official FBDG across different countries [11, 14, 18, 19]. In the study conducted by James-Martin et al. [11], which evaluated compliance with the 16 FAO principles for a sustainable healthy diet [20], France scored poorly because it did not numerically consider environmental criteria while setting their dietary guidelines and omitted other principles. In contrast, the Belgian guidelines received the best score for the consumer official document.

In another report, a climate change score was assigned to the guidelines from 93 countries [18]. Here again, Belgian dietary guidelines received the best score (84/100), while the French ones were rated lower (51/100). The latter score was primarily undermined by the lack of any reference to substituting animal products. Finally, the guidelines regarding animal products, and hence the scope for consuming these food groups, seem to be a crucial factor in ensuring the sustainability of appropriate diets, especially in environmental terms.

### Strengths and limitations

Our study has a few limitations. We acknowledge that the percentage of women in our study sample is higher than in the general population, but we are considering a weighting process to reflect an average individual. Additionally, as recruitment is based on voluntary participation, there is likely a bias related to the non-representativeness of the population and its more health-conscious profile. These are the significant biases inherent in the NutriNet-Santé study and other cohorts founded on voluntary recruitment [62]. Several studies aiming to characterize the population have been conducted [28]. Because the individuals who participated in the study were all volunteers, who were presumably more interested in nutritional matters, their initial diets before optimization modeling were already quite rich in plant-based foods compared to what is typically observed in the general population. This has probably led to higher 99th percentile values than those of a representative sample. In addition, although the FFQ was validated, self-reported data are prone to measurement error, and consumption may have been underestimated, as illustrated by the difference between energy requirement and energy intake in observed diets. The LCA only considered the production stage, as data for the entire system (from farm to fork) were not accessible for organic systems. However, whether for organic or standard/conventional farming systems, the LCA, which has rarely been considered before, indicates that the production phase has the highest emissions [63].

Additionally, it has been established that the LCA may misrepresent some ecosystem services, particularly for agroecological practices [64]. It would also be valuable to consider other environmental indicators, as discussed above, as well as consequential LCA. Here, the consequences regarding the reshaping of agricultural practices and mitigation associated with the lower production of animal products are not considered. Finally, concerning acceptability constraints, they were defined by upper bounds set at the weighted 99th percentile values of each food group based on the weighted distribution in the sample. Due to the lack of specific data on acceptability, these upper bounds reflect the overall feasibility based on current consumption levels. An upper threshold was established to cap extreme or unrealistic consumption levels while allowing room to maneuver for change.

Our study has many strengths. When modeling diets, we considered many nutrient reference values, including bioavailability for iron and zinc, as well as cultural “acceptability,” which corresponds to the apparent feasibility of the solutions. We also used recent, reliable data from the GBD as a proxy for the potential impact of the diet on health.

## Conclusion

In conclusion, this study highlights specific dietary adjustments that can significantly reduce the environmental footprint of diets while also providing health benefits. According to scientific literature, dietary changes alone could reduce environmental impact by up to 80%. A key adjustment involves redefining the role of meat in dietary guidelines, including the introduction of thresholds for different types of meat, with a particular focus on ruminant meat. To achieve truly sustainable diets, a multidisciplinary approach is essential. This approach should consider a range of factors beyond greenhouse gas emissions, addressing various environmental, health, and socio-economic issues.

## Electronic supplementary material

Below is the link to the electronic supplementary material.

Supplementary Material 1

## Data Availability

Data described in the manuscript, code book, and analytic code will be made available upon request pending application and approval. Researchers from public institutions can submit a collaboration request including information on the institution and a brief description of the project to collaboration@etude-nutrinet-sante.fr. All requests will be reviewed by the steering committee of the NutriNet-Santé study. If the collaboration is accepted, a data access agreement will be necessary and appropriate authorizations from the competent administrative authorities may be needed. In accordance with existing regulations, no personal data will be accessible.

## Abbreviations

FBDG: Food-based dietary guidelines
GBD: Global Burden of Diseases
GHGe: Greenhouse gas emissions
P: Percentile
TMREL: Theoretical minimum-risk exposure level
