# Supplementary material for: To be climate-friendly, food-based dietary guidelines must include limits on total meat consumption – modeling from the case of France
Source: Int J Behav Nutr Phys Act. 2025 Jul 9;22:95. doi: 10.1186/s12966-025-01786-9 (PMC12239361; doi:10.1186/s12966-025-01786-9)
Supplement: Supplementary file 1 — Supplementary Material 1 [file 12966_2025_1786_MOESM1_ESM.docx]

**Supporting Information**

**To be climate-friendly, food-based dietary guidelines must include limits on meat consumption – modelling from the case of France**

# Emmanuelle Kesse-Guyot^1^, Julia Baudry^1^, Justine Berlivet^1^, Elie Perraud^1^, Chantal Julia^1, 2^Mathilde Touvier^1^, Benjamin Allès, Denis Lairon^2^, Serge Hercberg^1^, Hélène Fouillet^3^, Philippe Pointereau^4^, François Mariotti^3^

^1^ Université Sorbonne Paris Nord and Université Paris Cité, INSERM, INRAE, CNAM, Center of Research in Epidemiology and StatisticS (CRESS), Nutritional Epidemiology Research Team (EREN), 93017 Bobigny, France

^2^ Public Health Department, Avicenne Hospital (Assistance Publique- Hôpitaux de Paris (AP-HP), Bobigny, France

3 AixMarseille Université, Inserm, INRAE, C2VN, 13005 Marseille, France

^4^ Université Paris-Saclay, AgroParisTech, INRAE, UMR PNCA, 91120, Palaiseau, France

^5^ Solagro, 75, Voie TOEC, CS 27608, F-31076 Toulouse Cedex 3, France

**Correspondence**: Email: [emmanuelle.kesse-guyot@inrae.fr](mailto:emmanuelle.kesse-guyot@inrae.fr)

Equipe de Recherche en Epidémiologie Nutritionnelle (EREN)

SMBH Université Sorbonne Paris Nord, 74 rue Marcel Cachin, 93017 Bobigny, France

[Emmanuelle Kesse-Guyot^1^, Julia Baudry^1^, Justine Berlivet^1^, Elie Perraud^1^, Chantal Julia^1, 2^Mathilde Touvier^1^, Benjamin Allès, Denis Lairon^2^, Serge Hercberg^1^, Hélène Fouillet^3^, Philippe Pointereau^4^, François Mariotti^3^ 1](#_Toc191392993)

[Supplemental Method 1: PNNS-GS2 computation 2](#_Toc191392994)

[Supplemental Method 2:Environmental data 3](#_Toc191392995)

[Supplemental Method 3:Iron bioavailability 4](#_Toc191392996)

[Supplemental Method 4: Zinc bioavailability 4](#_Toc191392997)

[Supplemental Method 5: Health Risk Score computation 4](#_Toc191392998)

[Supplemental Table 1: Components, scoring and weighting used for adherence score, i.e. sPNNS-GS2, computation 6](#_Toc191392999)

[Supplemental Table 2: Nutritional constraints used in the optimization models 7](#_Toc191393000)

[Supplemental Table 3: Characteristics of the Sample (N=29,413) by weighted quantiles of PNNS-GS2 scores^1^ 8](#_Toc191393001)

[Supplemental Table 4: Consumption of food groups in GHG-imposed scenarios^1^ 10](#_Toc191393002)

[Supplemental Table 5: Consumption of food groups (g/d) in GHG-imposed scenarios - sensitivity analysis using the 95th percentile as the maximum of food group consumption)^1^ 12](#_Toc191393003)

[Supplemental Figure 2: Contribution of food groups to nutrient intake in GHG-imposed scenarios^1,2^ 14](#_Toc191393004)

# Supplemental Method 1: PNNS-GS2 computation

In March 2017, as part as the developmentof the fourth Programme National Nutrition Santé (2017-2021), the Haut Conseil de Santé Publique (HCSP) published a report updating the 2001 PNNS recommendations (1) based on scientific literature about the relationships between diet and long-term health and a model created by the Agence nationale de sécurité sanitaire de l'alimentation, de l'environnement et du travail (2). These new recommendations provide dietary guidelines, with 6 adequacy guidelines ("fruits and vegetables", "nuts", "legumes", "whole foods", "milk and dairy products", and "fish and seafood") and 6 moderation guidelines ("meat", "processed meat", "added fats", "sugary products", "beverages", and "salt"). Physical activity recommendations are not addressed by the HCSP. The nutrition experts who were involved in developing the guidelines defined the thresholds and corresponding scores. These thresholds and related scores are defined so asthat following the guidelines is associated with one point, whereas not following them is scored zero points for healthy foodss. To increase the power of discrimination, half-points are allocated in a linear fashion above the guideline thresholds. However, an exception was made for milk and dairy products, and fish. As, the relationship between these foods and health is non-linearpoints allocated points form a parabolic relationship (3).

The PNNS-GS2 emphasizes on the distinction between malus components (unhealthy food thought to be avoided, which have a negative moderation score, e.g. salt) and bonus components (healthy foods considered beneficial, which have a positive adequacy score, e.g. legumes).

All details regarding the scoring system are shown in **Supplemental Table 1.**

# Supplemental Method 2:Environmental data

The methodology used for the assessment of the environmental impacts in the NutriNet-Santé study has been extensively described elsewhere (4). The data pertaining to the environmental impacts of raw agricultural products have also been disclosed elsewhere (4).

**Evaluation of the agricultural production stage**

Data sources and perimeter of analysis

Environmental data were derived from the French diagnostic tool DIALECTE (5). DIALECTE is a comprehensive tool developed by Solagro which aims to describe farming systems and to assess the environmental performance of farms using a global approach at farm scale. To date, DIALECTE database includes information from 2,086 farms with various agricultural production systems, in particular organic farming, throughout France. A total of 46% of farms included in the DIALECTE database followed certified organic practices.

Information collected by DIALECTE allowed estimating the environmental impacts at the farm level of 60 raw agricultural products using the Life Cycle Assessment (LCA) method. Here, the perimeter of LCA was limited to the agricultural production step. Of note, the upstream processes were included in the assessment such as the production of inputs or energy provision. Conditioning, transport, processing, storage and recycling were excluded from the analysis. This limitation should be considered as relative since most environmental impacts generally occur from ‘the agricultural phase’ (6,7). This is less true for alcoholic beverages as regards energy consumption and CO2 emissions since they require high level of processing and bottling, or for fruit and vegetables transported by plane.

The choice of DIALECTE pertained to its great diversity of data based on real French farms from various types of farming systems. Apart from the Agribalyse project (8) in which organic data are scarce, to our knowledge, DIALECTE is to date the only French database that covers such a large panel data for both organic and conventional agricultural products, making it possible the calculation of LCA-based indicators and for a whole diet.

Assessment of environmental indicators at farm scale

- Cumulative energy demand (CED)

Cumulative energy demand (in MJ) included consumption of renewable and unrenewable energy as defined by the CED method (9), commonly used in LCA. The calculation system used was based on the Dia’terre^®^ instrument (10). The use of renewable energies is limited at farm level, as a very low percentage of farms in DIALECTE have solar dryers, water panels or biogas plant which reduce the consumption of unrenewable energies.

The following table shows the different cumulative energy demand items and the nature of the data used. Most of the energy demands positions were evaluated at the farm level. Allocations (with generic energy demand coefficient) were computed to share the energetic consumption between vegetal production and livestock building.

Cumulative energy demand posts and calculation method by production

| **Cumulative energy demand posts** | **Survey data** | **Attribution** |
| --- | --- | --- |
| *Directs energy (without irrigation)* | |  |
| Fuel^1^ (Fuel, lubricants, diesel and petrol) | Data at farm level | Allocation of energy consumption in proportion to the theoretical consumption of crops and livestock |
| Gas (propane, butane, natural gas) |  |  |
| Electricity |  |  |
| *Direct energy for irrigation* | |  |
| Fuel^1^ | Data at farm level | Prorated calculation of attribution in function of theoretic consumption between vegetable production and livestock building |
| Electricity^1^ |  |  |
| *Indirect energy* | | |
| Livestock and veterinary products | Data at farm level | Non-included |
| Mechanization |  | Prorated calculation of attribution for vegetable production, in function of land occupation |
| Building (<30 years) |  |  |
| Plastic sheeting |  |  |
| Preservatives or silage additives |  |  |
| Feed |  | Prorated calculation of attribution for animals in function of the consumption of forage and concentrates |
| Fertilizer | Data at crop level | N/A |
| Phytosanitary products |  |  |

^1^Including consumption for third parties. N/A, Not applicable

- Greenhouse gas emissions (GHGEs)

The GHGE referred to the definition established by the Intergovernmental Panel on Climate Change, *i.e.* the quantity of three GHGs (carbon dioxide CO_2_, methane CH_4_ and nitrous oxide N_2_O), weighted by their 100-year global warming potential, in kg CO_2_-eq. Their calculation was based on the methodology developed in the tool Dia’terre^®^(10).

The following table shows the different posts of GHGEs taken into account in the LCA. Allocations with generic energy demand coefficient were used to share the emissions between vegetable production and livestock building.

Point of GHG emissions and calculation method by production

| **Source of GHGEs** | **Survey data** | **Allocation** |
| --- | --- | --- |
| *Direct energy* | | |
| Direct energy without irrigation  (fuel, gas, electricity) | Data at farm level | Prorated calculation in function of theoretic consumption between vegetable production and livestock building |
| Direct energy for irrigation  (fuel, electricity) | Data at farm level | Prorated calculation in function of water consumption by crop |
| *Indirect energy* | | |
| Mechanization, plastics, building (< 30 years), preservatives silage and veterinary products | Data at farm level | Prorated calculation for vegetable production, in function of land occupation. |
| Fertilizers and pesticides | Data at crop level | N/A |
| Feed | Data at farm level | Prorated calculation for animals in function of the consumption of forage and concentrates. |
| **Diffuse emissions** | | |
| Enteric fermentation | Data by domestic animal category | N/A |
| Direct emission of N_2_O (Application of nitrogen fertilizers and manure, nitrogen excreted during grazing, mineralization of crop residues. | Data at crop level |  |
| Indirect emission of N_2_O (atmospheric deposition, runoff and leaching of reactive nitrogen) |  |  |
| **Carbone storage in soil and in trees** |  | Non-included |

N/A, Not applicable

- Land occupation

Land occupation corresponded to the area required to produce raw agricultural products, without taking into account the duration of the land use. It is expressed as the inverse of yield, in m^2^.kg^-1^. This indicator differs from the LCA indicator *land use,* expressed in m^2^.kg^-1^.year, which reflects the surface of land occupied over a fixed period of time. Both indicators are similar when the production period is one year, which corresponds to most agricultural production cycles.

For 29 types of vegetables, the impacts were calculated using the average yield between 2010 and 2015 from the agricultural annual French statistics, rather than survey data. Indeed, most vegetable are produced on small areas and yield calculation over one hectare may lead to discrepancies. As the French statistics provided data for conventional farming only, we used a rebate coefficient for organic farming of 23% for all vegetable crops, excepted for strawberries, 41% and salads 14% (11). Also, for vegetable crops, impacts per hectare were computed depending on the mean of production: heated or cold greenhouses or field grown (11).

For animal production, the indicator was calculated as the sum of the land areas needed for pasture and feed production, including purchased feed while neglecting areas related to livestock farm facilities.

Aggregation for particular products

Some agricultural products available in DIALECTE but not in BioNutriNet database were aggregated into one single product reference (see table below) by using a ponderation on the Annual Agricultural Survey from the French Ministry of Agriculture (12).

Examples of aggregation of some specific agricultural products

| Aggregated agricultural product | DIALECTE agricultural product | Proportion |
| --- | --- | --- |
| Olive | Black olive | 0.5 |
|  | Green olive | 0.5 |
| Chicken | Standard chicken | 0.88 |
|  | Labelled chicken | 0.12 |
| Bovine meat | From cattle rearing and fattening farms | 0.66 |
|  | From dairy cattle | 0.34 |
| Sheep meat | Sheep fattening | 0.9 |
|  | Dairy Sheep | 0.1 |

Moreover, tomatoes produced under heated greenhouses were considered as ‘cherry tomatoes’ or ‘fresh tomatoes’ and tomatoes produced in open fields as ‘cooked tomatoes’ and ‘tomato pulp’.

Determination of the reference value

Using the methodology described above, we obtained environmental impacts for 62 raw agricultural products from 2,086 farms surveyed with DIALECTE, of which 46% were conducted according to organic principles. Regarding organic farming, the number of cases available ranged from 2 (duck) to 355 (beef meat) (Interquartile range (IQR): 34; 348). Concerning conventional farming, the number of available data ranged from 1 (nuts) to 408 (soft wheat) (IQR: 40; 348). Medians were taken as central values since outliers are relatively common in this type of survey.

For products with a high contribution to the diet and for which data were not available in DIALECTE, such as seafood (N=6), honey (N=1), tropical products (N=12) and for some products with few references in DIALECTE (N=13), impacts were assessed with published literature data, and Agribalyse® data in particular (13).

Finally, the three environmental indicators were calculated for 92 raw agricultural products, conventional and organic, 60 from DIALECTE and 32 from literature searches.

**Conversion from raw agricultural products to food items**

As our goal was to assess the environmental impacts of diets, we conducted a set of conversions to obtain data at the consumer level.

Conversion from raw agricultural products to ingredients

As a first step, to obtain values for each indicator and ingredient from agricultural raw products, economic attributions by co-products were applied followed by the allocations of mass, cooking, and edibility coefficients in order to obtain indicator values for the ingredient as consumed, using data from literature and NutriNet-santé composition database (for the allocations of cooking and edibility coefficients). The same conversion factors were used for organic and conventional products.

Data were unavailable for 59 minor ingredients which accounted for more than 5% in at least one recipe (*e.g.* tropical areas, certain alcoholic beverages, grains or yeast). Their environmental impacts were considered as null.

Conversion from ingredients to food items

Each of the 264 items of the Org-FFQ (Organic Food Frequency Questionnaire) was composed of sub-items (or foods). For example, the item pasta was composed of many sub-items (vermicelli, pasta, Chinese noodles, egg pasta, fresh pasta, and egg and fresh pasta spinach flavor). The composition of each item was the result of the multiplication of gender-specific consumption frequency of each sub-item (determined using the 24-h dietary record tool of NutriNet-Santé) by the ingredients constituting the sub-item.

Overall, a total of 766 ingredients are components of the 264 food items of the Org-FFQ. For feasibility reasons, the environmental impacts of the ingredients which accounted for at least 5% of the recipe were computed, corresponding to 442 ingredients.

About 75% of food items had more than 98.8% of their composition covered by the 442 ingredients. When an ingredient was missing for a given item, environmental impacts were ‘standardized’ for 100g. The ingredients especially concerned by the standardization were fish products, jam, cheeses and dressings.

Finally, we obtained environmental impacts for most of the 264 food items in organic and conventional forms. No data were available for 22 items for CED, 21 items for GHGE and 25 items for land occupation. Missing data included the following food items: water, certain alcoholic beverages, tropical fruit or vegetables, grains and certain oils.

# Supplemental Method 3:Iron bioavailability

In order to consider iron bioavailability, heme and non-heme iron were considered (14).

The rate of absorption for heme iron was calculated as (15) :

$$\text{Log Absorption }\left( \text{\%} \right)\text{= 1.9897 – 0.3092 × log (SF) }$$

where SF is serum ferritin (μg/L). We considered a stringent situation by setting serum ferritin at 15 mg/L

The rate of absorption for non-heme iron was calculated as (16) :

$\text{Ln Absorption }\left( \text{\%} \right)\text{= }\text{6.294 – 0.709 }\text{ln}\text{ }\left( \text{SF} \right)\text{+ 0.119 ln }\left( \text{VitC} \right)\text{+ 0.006 ln }\left( \text{MFP + 0.1} \right)\text{ } \text{-0.055}\ln\left( \text{T+0.1} \right) \text{-0.247}\ln\left( \text{Phy} \right)\text{-0.137}\ln\left( \text{Ca} \right)\text{-0.083 ln (NHI)}$

where SF is serum ferritin (μg/L) which was also set at 15 mg/L, VitC is vitamin C intake (mg), MFP corresponds to consumption of meat, fish, and poultry (g), T is tea intake (as number of cups), Phy is phytate intake (mg), Ca is calcium intake (mg), and NHI is non-heme iron intake (mg).

# Supplemental Method 4: Zinc bioavailability

For zinc absorption, we used the equation developed and updated by Miller and al. (17)

$$BZ (mmol/d)=0.5 \left\{ 0.069 \left( 1+\frac{PhyI (1-0.017CI)}{0.44} \right)+ 0.084 + ZI (1+ 0.012PI) -\sqrt{\left( \left( 0.069(1+\frac{PhyI(1-0.017 CI)}{0.44}) + 0.084 + ZI (1+0.012PI \right)^{2}- 4\times0.084 ZI (1+0.012PI \right)} \right\}$$

Where BZ is bioavailable zinc and PhyI, CI, ZI, and PI are phytates, calcium, zinc and proteins daily intakes respectively. All variables are in units of mmol/d except protein which is in g/d.

# Supplemental Method 5: Health Risk Score computation

The Global burdent of diseases (GBD) study by the IHME aimed to identify health challenges worldwide (18). It defines theoretical minimum-risk exposure levels (TMREL) for unhealthy (red meat, processed meat and sweetened beverages) and healthy (wholegrain products, fruits, vegetables, pulses, nuts and seeds, and milk) food groups. The levels aim to estimate recommended target intakes for each of these groups. The study provides the DALYs associated with excessive or insufficient consumption of these food groups. A Health Risk Score (HRS) evaluates the health risks associated with a given diet (19). HRS is minimal when the unhealthy food group intake is lower or equal to TMREL value and healthy food group intake is greater or equal to TMREL value. The distance to each food group target is weighted by the relative importance of reaching this target compared to others in terms of DALYs. HRS is maximal when the diet is at the maximal possible distance from each TMREL value.

The following formula is as follows:

$$\text{HRS}=100*\left[ \sum_{i=1}^{3} \left\{ \frac{Cons\text{ }\left( i \right)}{Mean\_pop (i)}\times\frac{DALYs\text{ }\left( i \right)}{DALYs\text{ }\left( all \right)} \right\} + \sum_{j=1}^{6} \left\{ max\left( \frac{TMREL (j) - Cons\text{ }\left( j \right)}{TMREL (j)}; 0 \right)\times\frac{DALYs\text{ }\left( j \right)}{DALYs\text{ }\left( all \right)} \right\} \right]$$

*Where:*

- i: unhealthy food groups to be limited (red meat, processed meat and sweetened beverages)
- j: healthy food groups to be promoted (wholegrain products, fruits, vegetables, legumes, nuts and seeds, milk)
- Cons: consumption of the item i or j
- Mean_pop (i): average consumption of the food group i (g/d) in the French population
- TMREL (j): TMREL value for the food group j (g/d)
- DALYs (i), DALYs(j): DALYs associated with over- and under-consumptions of the food groups i and j respectively (years)
- DALYs (all): sum of all DALYs(i) and DALYs(j)

The TMREL and corresponding DALYs values used were the following:

|  |  | TMREL^1^ (g/d) | DALYs^2^ (y) |
| --- | --- | --- | --- |
| Healthy foods | Wholegrain products | 150 | 42 392 |
|  | Fruits | 325 | 29 643 |
|  | Legumes | 95 | 20 489 |
|  | Vegetables | 300 | 12 432 |
|  | Nuts and seeds | 14.5 | 7 885 |
|  | Milk | 430 | 6 248 |
| Unhealthy foods | Red meat | 0 | 49 386 |
|  | Processed meat | 0 | 20 634 |
|  | Sweetened beverages | 0 | 5 896 |
| Total |  |  | 195 006 |

^1^According to the most recent (2019) estimates from the GBD, TMREL values are global estimates corresponding to a mean energy intake of 2,300 kcal (18).

^2^ DALYs values associated with excessive/insufficient consumptions of unhealthy/healthy foods are available at the Global Health Data Exchange website (<http://ghdx.healthdata.org/gbd-results-tool>).

**Supplemental Table 1: Components, scoring and weighting used for adherence score, i.e. sPNNS-GS2, computation**

| **Dietary components** | **Recommendation** | **Criteria** | **Score** |
| --- | --- | --- | --- |
| Fruit and | At least 5 serv/d, | [0 - 3.5[ | 0 |
| vegetables | with 1 max as juice and 1 | [3.5 - 5[ | 0.5 |
| (weight=3) | max as dried | [5 - 7.5[ | 1 |
|  |  | ≥7.5 | 2 |
| Nuts | A handful/d | 0 | 0 |
| (weight=1) |  | ]0 – 0.5[ | 0.5 |
|  |  | [0.5- 1.5[ | 1 |
|  |  | ≥1.5 | 0 |
| Pulses | At least 2 serv/w | 0 /w | 0 |
| (weight=1) |  | ]0-2[ /w | 0.5 |
|  |  | ≥2 /w | 1 |
| Whole-grain | Every day | 0 | 0 |
| Food |  | ]0 - 1[ | 0.5 |
| (weight=2) |  | [1 - 2[ | 1 |
|  |  | ≥2 | 1.5 |
| Milk and | 2 serv/d | [0 - 0.5[ | 0 |
| dairy products |  | [0.5 - 1.5[ | 0.5 |
| (weight=1) |  | [1.5 - 2.5[ | 1 |
|  |  | ≥2.5 | 0 |
| Red meat | Limit consumption | ≥750 g/w | -2 |
| (weight=2) |  | [500 - 750[ g/w | -1 |
|  |  | [0 - 500[ g/w | 0 |
| Processed meat | Limit consumption | ≥300 g/w | -2 |
| (weight=3) |  | [150 - 300[ g/w | -1 |
|  |  | [0 - 150[ g/w | 0 |
| Fish and | 2 serv/w | [0 - 1.5[serv /w | 0 |
| Seafood |  | [1.5 - 2.5[serv /w | 1 |
| (weight=2) |  | [2.5 - 3.5[serv /w | 0.5 |
|  |  | ≥3.5 serv /w | 0 |
| Added fat | Avoid overeating | >16% of EIWA ^c^ | 0 |
| (weight=2) |  | ≤16% of EIWA | 1.5 |
| Sugary foods | Limit consumption | ≥15% of EIWA | -2 |
| (weight=3) |  | [10-15[% of EIWA | -1 |
|  |  | <10 % of EIWA | 0 |
| Sweet drinks | Limit consumption | ≥ 750mL mL/d | -2 |
| Beverages |  | [250 - 750[ mL/d | -1 |
| (weight=3) |  | ]0 - 250[ mL/d | -0.5 |
|  |  | 0 mL/d | 0 |
| Alcoholic | Limit consumption | >200 g/d | -2 |
| beverages |  | ]100-200] g/d | -1 |
| (weight=3) |  | ]0-100] g/d | 0 |
|  |  | 0 g/d | 0.5 |
| Salt | Limit consumption | >12 g/d | -2 |
| (weight=3) |  | ]10-12] g/d | -1 |
|  |  | ]8-10] g/d | -0.5 |
|  |  | ]6-8] g/d | 0 |
|  |  | ≤6 g/d | 1 |

Abbreviations: d: day; EIWA: Energy intake without alcohol; w: week;

# Supplemental Table 2: Nutritional constraints used in the optimization models

|  | **Type of reference** | **Men** |  | **Women** |  | **Average individual^1^** |  |
| --- | --- | --- | --- | --- | --- | --- | --- |
|  |  | **Lower reference** | **Upper reference** | **Lower reference** | **Upper reference** | **Lower reference** | **Upper reference** |
| EI | - | ER - 8% | ER + 8% | ER - 8% | ER + 8% | ER - 8% | ER + 8% |
| Protein | RDA | 0.83 × bw g | 2.3 × bw g | 0.83 × bw g | 2.3 × bw g | 0.83 × bw g | 2.3 × bw g |
| Vitamin A | RDA | 750 µg | 3000 µg | 650 µg | 3000 µg | 700 µg | 3000 µg |
| Vitamin B1 | RDA | 0.42 µg /kcal | - | 0.42µg /kcal | - | 0.42µg /kcal | - |
| Vitamin B2 | RDA | 1.60 mg | - | 1.60 mg | - | 1.60 mg | - |
| Vitamin B3 | RDA | 6.7 µg /kcal | 900 µg | 6.7 µg /kcal | 900 µg | 6.7 µg /kcal | 900 µg |
| Vitamin B5 | AI | P5 mg | - | P5 mg | - | Weighted P5 | - |
| Vitamin B6 | RDA | 1.7 mg | 25 mg | 1.6 mg | 25 mg | 1.65 mg | 25 mg |
| Vitamin B9 | RDA | 330 µg | - | 330 µg | - | 330 µg | - |
| Vitamin B12 | RDA | 4 µg | - | 4 µg | - | 4 µg | - |
| Vitamin C | RDA | 110 mg | - | 110 mg | - | 110 mg | - |
| Vitamin E | AI | P5 g | - | P5 g | - | Weighted P5 | - |
| Vitamin K | AI | P5 µg | - | P5 µg | - | Weighted P5 | - |
| Calcium | RDA | 950 mg | 2500 mg | 950 mg | 2500 mg | 950 mg | 2500 mg |
| Copper | AI | P5 g | 5 g | P5 g | 5 g | Weighted P5 | 5 g |
| Bioavailable Iron^2^ | RDA | 1.10 mg | - | 1.10/1.16 mg | - | 1.13 mg |  |
| Iodine | RDA | 150 µg | 600 µg | 150 µg | 600 µg | 150 µg | 600 µg |
| Magnesium | AI | P5 g | - | P5 g | - | Weighted P5 | - |
| Manganese | AI | P5 g | - | P5 g | - | Weighted P5 | - |
| Phosphorus | RDA | 550 mg | - | 550 mg | - | 550 mg | - |
| Potassium | RDA | 3500 mg | - | 3500 mg | - | 3500 mg | - |
| Selenium | RDA | 70 µg | 300 µg | 70 µg | 300 µg | 70 µg | 300 µg |
| Sodium | RDA | 1500 mg | 2300 mg | 1500 mg | 2300 mg | 1500 mg | 2300 mg |
| Bioavailable zinc | RDA | 1.6 mg |  | 1.3 mg |  | 1.45 mg |  |
| SFA | RDA | - | 12% EI | - | 12% EI | - | 12% EI |
| Linoleic acid | RDA | 4% EI | - | 4% EI | - | 4% EI | - |
| ALA | RDA | 1% EI | - | 1% EI | - | 1% EI | - |
| LA / ALA | RDA | - | 5 | - | 5 | - | 5 |
| EPA+DHA | RDA | 0.5 g | - | 0.5 g | - | 0.5 g | - |
| Sugar without lactose | RDA | - | 100 g | - | 100 g | - | 100 g |
| Fiber | RDA | 30 g | - | 30 g | - | 30 g | - |

Abbreviations: AI, adequate intake; ALA, alpha-linoleic acid; bw, body weight (kg); DHA, docosahexaenoic acid; EI, energy intake; EPA, eicosapentaenoic acid; ER, energy requirement; LA, linolenic acid; M^-^, non-menopausal; M^+^, menopausal; RDA, recommended Dietary Allowances; SFA, saturated fatty acids.

The upper value of RDA is the Tolerable Upper Intake Level

^1^In case of different references between men and women. The average individual was the weighted mean as follows: 50% men, 25% women M-, 25% M+.

^2^threshold corresponding to a deficiency prevalence ≤5%

^3^threshold corresponding to the deficiency cut-off

# Supplemental Table 3: Characteristics of the Sample (N=29,413) by weighted quantiles of PNNS-GS2 scores^1^

|  | Q1 | Q2 | Q3 | Q4 | Q5 | Ptrend^2^ |
| --- | --- | --- | --- | --- | --- | --- |
| **sPNNS-GS2** | -2.72 (2.24) | 0.52 (1.25) | 2.38 (1.06) | 4.23 (0.94) | 6.90 (1.34) | <0.001 |
| **Gender (%)** |  |  |  |  |  |  |
| Women | 50.2 | 50.4 | 49.8 | 49.8 | 50.2 | 0.83 |
| Men | 49.8 | 49.6 | 50.2 | 50.2 | 49.8 |  |
| **Age** | 54.26 (13.78) | 54.58 (14.09) | 54.68 (14.15) | 55.39 (13.85) | 56.02 (13.66) | <0.001 |
| **Education (%)** |  |  |  |  |  |  |
| < High-school diploma | 59.8 | 63.3 | 64.2 | 64.5 | 64.8 | <0.001 |
| High school diploma | 14.6 | 14.1 | 13.8 | 13.4 | 13.4 |  |
| Postgraduate | 25.6 | 22.6 | 22 | 22.1 | 21.7 |  |
| **Income^3^ (%)** |  |  |  |  |  |  |
| <900 € | 6.9 | 6.2 | 5.5 | 5.8 | 6.7 |  |
| 900 – 1,200 € | 5.6 | 4.3 | 4 | 3.6 | 4 |  |
| 1,200 – 1,800 € | 24.6 | 24 | 22.5 | 21.5 | 19.9 | <0.001 |
| 1,800 – 2,300 € | 15.6 | 15.2 | 16.2 | 16.4 | 15.6 |  |
| 2,300 – 2,700 € | 11.4 | 10.8 | 12.2 | 12 | 11.5 |  |
| 2,700 – 3,700 € | 19.1 | 20.1 | 19.3 | 20.3 | 20.2 |  |
| ≥3700 € | 12.2 | 14.2 | 15.6 | 15.2 | 16.5 |  |
| **Occupation status (%)** |  |  |  |  |  |  |
| Unemployed | 3.8 | 3.8 | 3.6 | 3.7 | 3.7 | 0.66 |
| Retired | 40.1 | 41.3 | 41.9 | 43 | 43.5 |  |
| Employees | 15.1 | 12.9 | 12 | 10.8 | 10.1 |  |
| Intermediate professions | 13 | 13.4 | 13.8 | 13.6 | 12.3 |  |
| Manger or intellectual | 20.1 | 20.9 | 22.3 | 22.3 | 23.1 |  |
| never employed | 5.9 | 5.7 | 4.7 | 4.8 | 5.4 |  |
| self-employed | 2.1 | 2 | 1.7 | 1.7 | 1.9 |  |
| **Marital status (%)** |  |  |  |  |  |  |
| Cohabiting | 90 | 90.2 | 88.3 | 87.7 | 86 | <0.001 |
| Single | 10 | 9.8 | 11.7 | 12.3 | 14 |  |
| **Smoking status (%)** |  |  |  |  |  |  |
| Former | 45.6 | 44.6 | 42.9 | 43.9 | 43.9 | <0.001 |
| Current | 14.8 | 10.8 | 10.6 | 9.2 | 6.4 |  |
| Never | 39.7 | 44.7 | 46.4 | 46.9 | 49.7 |  |
| **Physical activity (%)** |  |  |  |  |  |  |
| High | 34.9 | 34.2 | 34.6 | 37.3 | 39.7 | <0.001 |
| Medium | 32.7 | 34.4 | 36 | 35.5 | 36.9 |  |
| Low | 22.3 | 21 | 19.2 | 16.9 | 13.3 |  |
| **Body mass index (kg/m²)** | 25.64 (4.80) | 24.89 (4.55) | 24.45 (4.40) | 24.24 (4.23) | 23.57 (4.02) | <0.001 |
| **Energy intake (Kcal/d)** | 2614 (699) | 2214 (618) | 2009 (576) | 1849 (534) | 1725 (460) | <0.001 |
| **%organic food** | 0.19 (0.20) | 0.23 (0.23) | 0.27 (0.26) | 0.32 (0.28) | 0.41 (0.31) | <0.001 |
| **% plant protein** | 24.88 (7.86) | 28.69 (9.71) | 32.34 (12.04) | 36.28 (14.62) | 43.51 (17.91) | <0.001 |
| **GHGe(kgCO_2_eq/d)/1000 Kcal** | 2.54 (0.97) | 2.26 (0.96) | 2.07 (0.90) | 1.86 (0.85) | 1.60 (0.76) | <0.001 |
| **LO(m²)** | 17.10 (9.40) | 12.91 (7.36) | 10.71 (5.86) | 8.92 (4.75) | 7.25 (3.58) | <0.001 |
| **CED (MJ/d)** | 25.48 (9.04) | 20.32 (7.52) | 17.51 (6.33) | 15.44 (5.70) | 13.61 (4.68) | <0.001 |
| **GHGe (kgCO_2_eq/d)** | 6.64 (3.33) | 4.98 (2.63) | 4.09 (2.08) | 3.37 (1.73) | 2.68 (1.32) | <0.001 |
| **Monetary cost (€/d)** | 9.75 ( 3.27) | 8.29 ( 3.00) | 7.63 ( 2.86) | 7.22 ( 2.76) | 7.07 ( 2.66) | <0.001 |
| **Consumption (g/d)** |  |  |  |  |  |  |
| Alcoholic beverages | 219.69 (241.12) | 142.64 (170.88) | 119.69 (167.92) | 98.46 (160.71) | 62.92 (80.65) | <0.001 |
| Animal fat | 8.64 (8.63) | 6.83 (7.29) | 5.84 (6.18) | 5.21 (5.95) | 4.08 (5.09) | <0.001 |
| Beef | 73.81 (54.87) | 51.24 (43.02) | 40.22 (35.67) | 30.89 (30.08) | 21.82 (21.49) | <0.001 |
| Cereals | 178.34 (102.61) | 154.71 (99.67) | 139.65 (93.47) | 124.68 (93.85) | 105.36 (86.24) | <0.001 |
| Dairy products | 216.11 (154.38) | 199.94 (141.44) | 183.73 (135.30) | 170.12 (129.88) | 153.20 (125.64) | <0.001 |
| Eggs | 13.40 (13.66) | 11.63 (12.13) | 11.05 (12.16) | 10.37 (11.16) | 10.38 (11.91) | <0.001 |
| Fish | 57.35 (59.62) | 49.07 (42.76) | 46.47 (41.08) | 45.48 (43.33) | 40.35 (37.74) | <0.001 |
| Fruit | 227.59 (210.74) | 260.21 (252.42) | 267.60 (249.74) | 289.00 (244.69) | 367.33 (273.71) | <0.001 |
| Fruit juice | 100.62 (137.41) | 91.07 (115.56) | 88.91 (118.76) | 83.60 (112.83) | 73.82 (101.25) | <0.001 |
| Milk | 72.65 (147.88) | 68.13 (148.61) | 62.97 (134.33) | 53.23 (128.84) | 39.74 (111.29) | <0.001 |
| Nuts | 4.88 (11.17) | 5.92 (14.21) | 7.81 (17.35) | 8.76 (16.80) | 13.25 (19.69) | <0.001 |
| Offal | 3.12 (5.87) | 2.49 (9.16) | 1.90 (10.72) | 1.65 (3.88) | 1.25 (3.02) | <0.001 |
| Mixed dishes^4^ | 41.02 (62.32) | 33.79 (34.10) | 27.34 (22.40) | 23.92 (20.64) | 20.28 (19.69) | <0.001 |
| Other fat | 10.30 (10.07) | 8.24 (8.96) | 7.03 (8.22) | 6.02 (6.95) | 5.00 (6.73) | <0.001 |
| Pork | 95.55 (60.48) | 63.61 (40.49) | 45.25 (28.67) | 32.37 (21.87) | 20.95 (17.20) | <0.001 |
| Potatoes | 33.99 (29.43) | 27.00 (24.87) | 23.44 (25.30) | 20.57 (20.63) | 17.37 (17.30) | <0.001 |
| Poultry | 34.37 (30.76) | 26.99 (24.76) | 23.18 (22.75) | 20.29 (23.06) | 17.52 (23.14) | <0.001 |
| Pulses | 15.30 (23.61) | 15.37 (33.42) | 15.37 (25.32) | 16.96 (29.81) | 23.38 (41.88) | <0.001 |
| SFF^5^ | 94.96 (76.69) | 81.62 (60.03) | 71.93 (51.55) | 63.46 (44.72) | 53.63 (37.82) | <0.001 |
| Snack | 17.88 (26.43) | 12.07 (13.23) | 10.31 (12.91) | 8.05 (10.29) | 5.79 (8.53) | <0.001 |
| Sweet drinks^6^ | 85.55 (167.96) | 52.44 (122.41) | 40.44 (86.21) | 32.98 (82.91) | 21.82 (49.45) | <0.001 |
| Substitutes | 17.43 (86.41) | 22.57 (107.63) | 35.14 (133.74) | 47.21 (155.87) | 76.12 (176.15) | <0.001 |
| Vegetable fat | 27.20 (18.96) | 23.65 (17.00) | 22.00 (15.67) | 20.85 (15.14) | 18.99 (13.73) | <0.001 |
| Vegetables | 338.92 (225.09) | 344.02 (231.34) | 342.90 (253.72) | 348.97 (221.61) | 397.51 (243.20) | <0.001 |
| Wholegrain products | 38.81 (60.33) | 49.03 (72.69) | 59.12 (77.11) | 65.47 (78.41) | 78.43 (77.20) | <0.001 |

Abbreviations: CED Cumulative energy demand; GHGe, greenhouse gas emissions; LO, land use; SFF, Sweet and fat foods

^1^Values presented are means (SD) and are weighted on the individual weight to represent the average individual.

^2^Chi² test or Kruskal-Wallis test as appropriate

^3^Income per consumption unit per month

^4^Mixed dishes include sandwiches, dishes such as pizza, hamburger, ravioli, panini, salted pancake

^5^Sweet and fat foods (SFF) include croissants, pastries, chocolate, biscuits, milky desserts, ice cream, honey and marmalade, cakes, chips, salted oilseeds, salted biscuits

^6^Sweet drinks include fruit nectar, syrup, soda (with or without sugar)

# Supplemental Table 4: Consumption of food groups in GHG-imposed scenarios^1^

|  |  | M0 | M1 | M2 | M3 | M4 | M5 | M6 | M7 | M8 | M9 | M10 | M11 | M12 | M13 | M14 | M15 | M16 | M17 | M18 | M19 | M20 | M21 | M22 | M23 | M24 | M25 | M26 | M27 | M28 |
| --- | --- | --- | --- | --- | --- | --- | --- | --- | --- | --- | --- | --- | --- | --- | --- | --- | --- | --- | --- | --- | --- | --- | --- | --- | --- | --- | --- | --- | --- | --- |
| **Alcoholic beverages** | alcoholic beverages | 1 | 1 | 1 | 1 | 1 | 1 | 1 | 1 | 1 | 1 | 1 | 1 | 1 | 1 | 1 | 1 | 1 | 1 | 1 | 1 | 1 | 1 | 1 | 1 | 1 | 1 | 1 | 1 | 1 |
| **Animal fat** | butter, lard etc. | 0 | 0 | 0 | 0 | 0 | 0 | 0 | 0 | 0 | 0 | 0 | 0 | 0 | 0 | 0 | 0 | 0 | 0 | 0 | 0 | 0 | 0 | 0 | 0 | 0 | 0 | 0 | 0 | 0 |
| **Beef** | beef/lamb, veal | 0 | 0 | 1 | 6 | 10 | 15 | 20 | 24 | 29 | 33 | 38 | 43 | 47 | 52 | 56 | 61 | 65 | 70 | 70 | 70 | 70 | 71 | 71 | 71 | 71 | 71 | 71 | 71 | 72 |
| **Refined cereals** | breakfast cereals | 27 | 18 | 12 | 12 | 12 | 11 | 11 | 10 | 10 | 9 | 9 | 8 | 8 | 7 | 7 | 6 | 6 | 5 | 5 | 5 | 5 | 4 | 2 | 0 | 0 | 0 | 0 | 0 | 0 |
|  | refined bread | 0 | 22 | 33 | 33 | 32 | 32 | 32 | 32 | 33 | 34 | 35 | 36 | 36 | 37 | 38 | 39 | 40 | 42 | 42 | 42 | 42 | 45 | 46 | 46 | 36 | 26 | 16 | 0 | 0 |
|  | refined cereal | 155 | 169 | 179 | 178 | 177 | 176 | 176 | 174 | 173 | 172 | 171 | 170 | 169 | 168 | 167 | 166 | 165 | 165 | 165 | 165 | 165 | 167 | 187 | 206 | 217 | 227 | 239 | 265 | 248 |
| **Dairy products** | cheese | 63 | 62 | 62 | 62 | 62 | 62 | 62 | 61 | 61 | 60 | 60 | 59 | 59 | 58 | 58 | 57 | 57 | 56 | 56 | 56 | 56 | 56 | 58 | 59 | 61 | 62 | 62 | 63 | 63 |
|  | cottage cheese | 0 | 0 | 0 | 0 | 0 | 0 | 0 | 0 | 0 | 0 | 0 | 0 | 0 | 0 | 0 | 0 | 0 | 0 | 0 | 0 | 0 | 0 | 0 | 0 | 0 | 0 | 0 | 0 | 0 |
|  | petits suisses | 0 | 3 | 5 | 5 | 5 | 5 | 5 | 5 | 5 | 5 | 5 | 4 | 4 | 4 | 4 | 4 | 4 | 4 | 4 | 4 | 4 | 4 | 5 | 6 | 6 | 5 | 4 | 0 | 0 |
|  | yogurt | 0 | 0 | 0 | 0 | 0 | 0 | 0 | 0 | 3 | 6 | 8 | 11 | 13 | 16 | 18 | 20 | 22 | 24 | 24 | 24 | 24 | 22 | 9 | 0 | 0 | 0 | 0 | 0 | 0 |
| **Eggs** | eggs | 0 | 0 | 0 | 0 | 0 | 0 | 0 | 0 | 0 | 0 | 0 | 0 | 0 | 0 | 0 | 0 | 0 | 0 | 0 | 0 | 0 | 0 | 0 | 0 | 0 | 0 | 0 | 0 | 0 |
| **Fish** | crustacean | 2 | 1 | 1 | 1 | 1 | 1 | 1 | 1 | 1 | 1 | 1 | 1 | 1 | 1 | 1 | 1 | 1 | 1 | 1 | 1 | 1 | 1 | 1 | 1 | 1 | 1 | 1 | 1 | 0 |
|  | fat fish | 16 | 16 | 16 | 16 | 16 | 16 | 16 | 16 | 16 | 16 | 16 | 16 | 16 | 16 | 16 | 16 | 16 | 16 | 16 | 16 | 16 | 16 | 16 | 16 | 16 | 16 | 16 | 16 | 16 |
|  | other fish | 11 | 11 | 12 | 12 | 12 | 12 | 12 | 12 | 12 | 11 | 11 | 11 | 11 | 11 | 11 | 11 | 11 | 11 | 11 | 11 | 11 | 11 | 11 | 11 | 11 | 12 | 12 | 12 | 13 |
| **Fruit** | compote | 3 | 4 | 4 | 5 | 5 | 5 | 6 | 6 | 7 | 7 | 7 | 8 | 8 | 9 | 9 | 9 | 10 | 10 | 10 | 10 | 10 | 10 | 10 | 10 | 10 | 9 | 5 | 0 | 0 |
|  | dried fruits | 0 | 0 | 1 | 1 | 1 | 1 | 1 | 2 | 2 | 2 | 2 | 2 | 2 | 2 | 2 | 2 | 2 | 2 | 2 | 2 | 2 | 2 | 3 | 3 | 3 | 3 | 2 | 0 | 0 |
|  | other fruits | 263 | 203 | 166 | 165 | 164 | 164 | 163 | 164 | 166 | 167 | 168 | 169 | 170 | 171 | 172 | 173 | 174 | 173 | 173 | 173 | 173 | 169 | 175 | 180 | 193 | 216 | 209 | 238 | 244 |
|  | high vitamin C fruits | 280 | 317 | 342 | 336 | 331 | 326 | 321 | 316 | 311 | 305 | 300 | 295 | 290 | 285 | 280 | 275 | 269 | 269 | 269 | 269 | 269 | 272 | 299 | 330 | 361 | 392 | 423 | 423 | 423 |
| **Fruit juice** | 100%fruit juice | 29 | 54 | 69 | 71 | 73 | 75 | 77 | 78 | 79 | 80 | 81 | 82 | 83 | 84 | 85 | 86 | 87 | 89 | 89 | 89 | 89 | 95 | 115 | 135 | 150 | 150 | 150 | 150 | 150 |
| **Milk** | milk | 0 | 0 | 0 | 0 | 0 | 0 | 0 | 2 | 2 | 1 | 1 | 0 | 0 | 0 | 0 | 0 | 0 | 0 | 0 | 0 | 0 | 3 | 10 | 13 | 0 | 0 | 0 | 0 | 0 |
| **Nuts** | nuts | 15 | 15 | 15 | 15 | 15 | 15 | 15 | 15 | 15 | 15 | 15 | 15 | 15 | 15 | 15 | 15 | 15 | 15 | 15 | 15 | 15 | 15 | 15 | 15 | 15 | 15 | 15 | 15 | 15 |
| **Offal** | offal | 2 | 2 | 4 | 4 | 3 | 3 | 3 | 3 | 3 | 3 | 3 | 3 | 3 | 3 | 3 | 3 | 3 | 2 | 2 | 2 | 2 | 0 | 0 | 0 | 0 | 0 | 0 | 0 | 0 |
| **Other** | prepared dishes | 0 | 0 | 0 | 0 | 0 | 0 | 0 | 0 | 0 | 0 | 0 | 0 | 0 | 0 | 0 | 0 | 0 | 0 | 0 | 0 | 0 | 0 | 7 | 15 | 20 | 19 | 0 | 0 | 0 |
| **Other fat** | dressing sauces | 0 | 0 | 0 | 0 | 0 | 0 | 0 | 0 | 0 | 0 | 0 | 0 | 0 | 0 | 0 | 0 | 0 | 0 | 0 | 0 | 0 | 0 | 0 | 0 | 0 | 0 | 0 | 0 | 0 |
| **Pork** | pork (fresh) | 0 | 0 | 2 | 2 | 2 | 2 | 2 | 2 | 2 | 2 | 2 | 1 | 1 | 1 | 1 | 1 | 1 | 0 | 0 | 0 | 0 | 0 | 0 | 0 | 0 | 0 | 0 | 0 | 0 |
|  | processed meat | 0 | 0 | 0 | 0 | 0 | 0 | 0 | 0 | 0 | 0 | 0 | 0 | 0 | 0 | 0 | 0 | 0 | 0 | 0 | 0 | 0 | 0 | 0 | 0 | 0 | 0 | 0 | 0 | 0 |
|  | white jam | 0 | 0 | 0 | 0 | 0 | 0 | 0 | 0 | 0 | 0 | 0 | 0 | 0 | 0 | 0 | 0 | 0 | 0 | 0 | 0 | 0 | 0 | 0 | 0 | 0 | 3 | 9 | 14 | 9 |
| **Potatoes** | potatoes | 0 | 0 | 0 | 0 | 0 | 0 | 0 | 0 | 0 | 0 | 0 | 0 | 0 | 0 | 0 | 0 | 0 | 0 | 0 | 0 | 0 | 0 | 0 | 0 | 0 | 0 | 0 | 0 | 0 |
| **Poultry** | poultry | 11 | 40 | 51 | 50 | 48 | 47 | 45 | 44 | 43 | 42 | 41 | 40 | 39 | 38 | 37 | 35 | 34 | 36 | 36 | 36 | 36 | 39 | 45 | 51 | 61 | 75 | 91 | 117 | 121 |
| **Pulses** | pulses | 136 | 117 | 106 | 104 | 101 | 98 | 96 | 93 | 90 | 87 | 85 | 82 | 79 | 76 | 74 | 71 | 68 | 65 | 65 | 65 | 65 | 63 | 64 | 66 | 66 | 65 | 72 | 97 | 96 |
| **SFF**^2^ | croissant | 61 | 50 | 44 | 43 | 42 | 40 | 39 | 38 | 37 | 36 | 35 | 34 | 33 | 32 | 31 | 30 | 29 | 28 | 28 | 28 | 28 | 27 | 27 | 26 | 25 | 26 | 23 | 7 | 0 |
|  | sweet and fat products | 16 | 38 | 47 | 48 | 49 | 51 | 52 | 52 | 53 | 53 | 54 | 54 | 55 | 55 | 56 | 56 | 57 | 58 | 58 | 58 | 58 | 57 | 45 | 31 | 15 | 0 | 0 | 0 | 0 |
| **Snack** | Sandwiches | 0 | 0 | 0 | 0 | 0 | 0 | 0 | 0 | 0 | 0 | 0 | 0 | 0 | 0 | 0 | 0 | 0 | 0 | 0 | 0 | 0 | 1 | 9 | 17 | 23 | 28 | 34 | 34 | 49 |
|  | Salted biscuits and chips | 0 | 0 | 0 | 0 | 0 | 0 | 0 | 0 | 0 | 0 | 0 | 0 | 0 | 0 | 0 | 0 | 0 | 0 | 0 | 0 | 0 | 0 | 0 | 0 | 0 | 0 | 0 | 0 | 0 |
| **Sweet drinks** | sweet drinks | 0 | 0 | 0 | 0 | 0 | 0 | 0 | 0 | 0 | 0 | 0 | 0 | 0 | 0 | 0 | 0 | 0 | 0 | 0 | 0 | 0 | 0 | 0 | 0 | 0 | 0 | 0 | 0 | 0 |
| **Substitutes** | soya-based cheese | 2 | 4 | 5 | 5 | 5 | 5 | 5 | 5 | 5 | 5 | 5 | 5 | 5 | 5 | 5 | 5 | 4 | 5 | 5 | 5 | 5 | 5 | 5 | 5 | 5 | 5 | 5 | 5 | 5 |
|  | soya-based “dairy” products | 0 | 0 | 0 | 0 | 0 | 0 | 0 | 0 | 0 | 0 | 0 | 0 | 0 | 0 | 0 | 0 | 0 | 0 | 0 | 0 | 0 | 0 | 0 | 0 | 0 | 0 | 0 | 0 | 0 |
|  | soya-based “milk” | 73 | 16 | 0 | 0 | 0 | 0 | 0 | 0 | 0 | 0 | 0 | 0 | 0 | 0 | 0 | 0 | 0 | 0 | 0 | 0 | 0 | 0 | 0 | 0 | 0 | 0 | 0 | 0 | 0 |
|  | soya dring | 20 | 10 | 1 | 2 | 2 | 2 | 3 | 2 | 1 | 1 | 0 | 0 | 0 | 0 | 0 | 0 | 0 | 0 | 0 | 0 | 0 | 0 | 0 | 0 | 0 | 0 | 0 | 0 | 0 |
|  | plant-based substitutes | 0 | 0 | 0 | 0 | 0 | 0 | 0 | 0 | 0 | 0 | 0 | 0 | 0 | 0 | 0 | 0 | 0 | 0 | 0 | 0 | 0 | 0 | 0 | 0 | 0 | 0 | 0 | 0 | 0 |
| **Vegetable fat** | margarine | 16 | 14 | 13 | 13 | 13 | 13 | 13 | 13 | 13 | 13 | 13 | 13 | 13 | 13 | 13 | 13 | 13 | 13 | 13 | 13 | 13 | 12 | 12 | 12 | 13 | 14 | 15 | 21 | 22 |
|  | other oil | 0 | 0 | 0 | 0 | 0 | 0 | 0 | 0 | 0 | 0 | 0 | 0 | 0 | 0 | 0 | 0 | 0 | 0 | 0 | 0 | 0 | 0 | 0 | 0 | 0 | 0 | 0 | 0 | 0 |
|  | ALA-rich oil | 31 | 32 | 33 | 33 | 33 | 33 | 33 | 33 | 33 | 33 | 33 | 33 | 33 | 33 | 33 | 33 | 33 | 33 | 33 | 33 | 33 | 33 | 33 | 32 | 32 | 30 | 29 | 25 | 24 |
| **Vegetables** | soup | 0 | 0 | 0 | 0 | 0 | 0 | 0 | 0 | 0 | 0 | 0 | 0 | 0 | 0 | 0 | 0 | 0 | 0 | 0 | 0 | 0 | 0 | 0 | 0 | 0 | 0 | 0 | 0 | 0 |
|  | vegetables | 930 | 930 | 930 | 930 | 930 | 930 | 930 | 930 | 930 | 930 | 930 | 930 | 930 | 930 | 930 | 930 | 930 | 930 | 930 | 930 | 930 | 930 | 930 | 930 | 930 | 930 | 930 | 930 | 930 |
| **Wholegrain products** | whole bread | 165 | 147 | 138 | 139 | 140 | 142 | 142 | 142 | 142 | 142 | 142 | 142 | 142 | 142 | 142 | 142 | 141 | 139 | 139 | 139 | 139 | 137 | 120 | 105 | 103 | 101 | 102 | 105 | 100 |
|  | whole cereals | 50 | 31 | 19 | 20 | 20 | 20 | 20 | 20 | 21 | 21 | 22 | 22 | 22 | 23 | 23 | 23 | 24 | 23 | 23 | 23 | 23 | 21 | 17 | 13 | 14 | 16 | 13 | 0 | 22 |

Abbreviations: M, model; SFF, Sweet and fat foods

^1^M0 to M28 denote models imposing GHGe of 1.2 to 6.8 kgCO2eq/d by increments of 0.2

^2^Sweet and fat foods (SFF) include croissants, pastries, chocolate, biscuits, milky desserts, ice cream, honey and marmalade, cakes, chips, salted oilseeds, salted biscuits

# Supplemental Table 5: Consumption of food groups (g/d) in GHG-imposed scenarios - sensitivity analysis using the 95th percentile as the maximum of food group consumption)^1^

|  | M0 | M1 | M2 | M3 | M4 | M5 | M6 | M7 | M8 | M9 | M10 | M11 | M12 | M13 | M14 | M15 | M16 | M17 | M18 | M19 | M20 | M21 | M22 |
| --- | --- | --- | --- | --- | --- | --- | --- | --- | --- | --- | --- | --- | --- | --- | --- | --- | --- | --- | --- | --- | --- | --- | --- |
| Alcoholic beverages | 1 | 1 | 1 | 1 | 1 | 1 | 1 | 1 | 1 | 1 | 1 | 1 | 1 | 1 | 1 | 1 | 1 | 1 | 1 | 1 | 1 | 1 | 1 |
| Animal fat | 0 | 0 | 0 | 0 | 0 | 0 | 0 | 0 | 0 | 0 | 0 | 0 | 0 | 0 | 0 | 0 | 0 | 0 | 0 | 0 | 0 | 0 | 0 |
| Beef | 0 | 0 | 1 | 6 | 10 | 15 | 20 | 24 | 29 | 33 | 38 | 42 | 47 | 51 | 56 | 61 | 65 | 71 | 71 | 71 | 71 | 71 | 71 |
| Refined cereals | 169 | 181 | 181 | 179 | 178 | 177 | 176 | 175 | 174 | 173 | 172 | 171 | 170 | 169 | 168 | 167 | 166 | 165 | 165 | 165 | 165 | 184 | 203 |
| Dairy products | 65 | 53 | 53 | 53 | 53 | 52 | 52 | 52 | 51 | 51 | 50 | 50 | 49 | 49 | 50 | 53 | 56 | 47 | 48 | 47 | 47 | 50 | 53 |
| Eggs | 0 | 0 | 0 | 0 | 0 | 0 | 0 | 0 | 0 | 0 | 0 | 0 | 0 | 0 | 0 | 0 | 0 | 0 | 0 | 0 | 0 | 0 | 0 |
| Fish | 29 | 29 | 29 | 29 | 29 | 29 | 29 | 29 | 29 | 29 | 29 | 29 | 29 | 29 | 29 | 29 | 29 | 29 | 29 | 29 | 29 | 29 | 29 |
| Fruit | 596 | 581 | 571 | 572 | 573 | 575 | 576 | 577 | 579 | 580 | 582 | 583 | 584 | 586 | 587 | 587 | 587 | 596 | 596 | 596 | 596 | 623 | 634 |
| Fruit juice | 85 | 139 | 150 | 150 | 150 | 150 | 150 | 150 | 150 | 150 | 150 | 150 | 150 | 150 | 150 | 150 | 150 | 150 | 150 | 150 | 150 | 150 | 150 |
| Milk | 60 | 92 | 94 | 95 | 96 | 98 | 99 | 101 | 103 | 104 | 106 | 108 | 110 | 111 | 111 | 108 | 106 | 118 | 118 | 118 | 118 | 110 | 96 |
| Nuts | 15 | 15 | 15 | 15 | 15 | 15 | 15 | 15 | 15 | 15 | 15 | 15 | 15 | 15 | 15 | 15 | 15 | 15 | 15 | 15 | 15 | 15 | 15 |
| Offal | 2 | 2 | 6 | 6 | 6 | 6 | 6 | 5 | 5 | 5 | 5 | 5 | 5 | 5 | 5 | 5 | 5 | 0 | 0 | 0 | 0 | 0 | 0 |
| Mixed dishes^2^ | 0 | 0 | 0 | 0 | 0 | 0 | 0 | 0 | 0 | 0 | 0 | 0 | 0 | 0 | 0 | 0 | 0 | 0 | 0 | 0 | 0 | 0 | 0 |
| Other fat | 0 | 0 | 0 | 0 | 0 | 0 | 0 | 0 | 0 | 0 | 0 | 0 | 0 | 0 | 0 | 0 | 0 | 0 | 0 | 0 | 0 | 0 | 0 |
| Pork | 0 | 0 | 0 | 0 | 0 | 0 | 0 | 0 | 0 | 0 | 0 | 0 | 0 | 0 | 0 | 0 | 0 | 0 | 0 | 0 | 0 | 0 | 0 |
| Potatoes | 0 | 0 | 0 | 0 | 0 | 0 | 0 | 0 | 0 | 0 | 0 | 0 | 0 | 0 | 0 | 0 | 0 | 0 | 0 | 0 | 0 | 0 | 0 |
| Poultry | 4 | 39 | 44 | 42 | 41 | 40 | 38 | 37 | 35 | 34 | 32 | 31 | 29 | 28 | 27 | 25 | 24 | 27 | 27 | 27 | 27 | 30 | 38 |
| Pulses | 343 | 317 | 308 | 302 | 296 | 289 | 282 | 275 | 269 | 262 | 255 | 249 | 242 | 235 | 229 | 223 | 216 | 210 | 210 | 210 | 210 | 233 | 267 |
| SFF^3^ | 29 | 39 | 45 | 45 | 45 | 44 | 44 | 43 | 43 | 42 | 42 | 42 | 41 | 41 | 40 | 40 | 40 | 37 | 37 | 37 | 37 | 22 | 7 |
| Snack | 0 | 0 | 0 | 0 | 0 | 0 | 0 | 0 | 0 | 0 | 0 | 0 | 0 | 0 | 0 | 0 | 0 | 0 | 0 | 0 | 0 | 13 | 24 |
| Sweet drinks^4^ | 0 | 0 | 0 | 0 | 0 | 0 | 0 | 0 | 0 | 0 | 0 | 0 | 0 | 0 | 0 | 0 | 0 | 0 | 0 | 0 | 0 | 0 | 0 |
| Substitutes | 100 | 16 | 0 | 0 | 0 | 0 | 0 | 0 | 0 | 0 | 0 | 0 | 0 | 0 | 0 | 0 | 0 | 0 | 0 | 0 | 0 | 0 | 0 |
| Vegetable fat | 56 | 56 | 55 | 55 | 55 | 55 | 55 | 54 | 54 | 54 | 54 | 54 | 54 | 54 | 54 | 53 | 53 | 53 | 53 | 53 | 53 | 53 | 53 |
| Vegetables | 611 | 611 | 611 | 611 | 611 | 611 | 611 | 611 | 611 | 611 | 611 | 611 | 611 | 611 | 611 | 611 | 611 | 611 | 611 | 611 | 611 | 611 | 611 |
| Wholegrain products | 223 | 196 | 190 | 191 | 192 | 193 | 194 | 195 | 196 | 197 | 198 | 199 | 200 | 201 | 202 | 203 | 204 | 204 | 204 | 204 | 204 | 186 | 167 |

Abbreviations: M, model; SFF, Sweet and fat foods

^1^M0 to M28 denote models imposing GHGe of 1.2 to 6.8 kgCO2eq/d by increments of 0.2 under nutritional, dietary guidelines and acceptability constraints using the 95^th^ percentile as maximum of food group consumption

^2^Mixed dishes include sandwiches, dishes such as pizza, hamburger, ravioli, panini, salted pancake

^3^Sweet and fat foods (SFF) include croissants, pastries, chocolate, biscuits, milky desserts, ice cream, honey and marmalade, cakes, chips, salted oilseeds, salted biscuits

^4^Sweet drinks include fruit nectar, syrup, soda (with or without sugar)

**Supplemental Figure 1. Study sample selection**

29,413 participants had place of purchase data for estimation of diet cost

37,685 participants completed the Org-FFQ

37,305 participants had no missing covariates

34,453 participants were not living overseas

35,196 participants were not under/over-reporters

# Supplemental Figure 2: Contribution of food groups to nutrient intake in GHG-imposed scenarios^1,2^


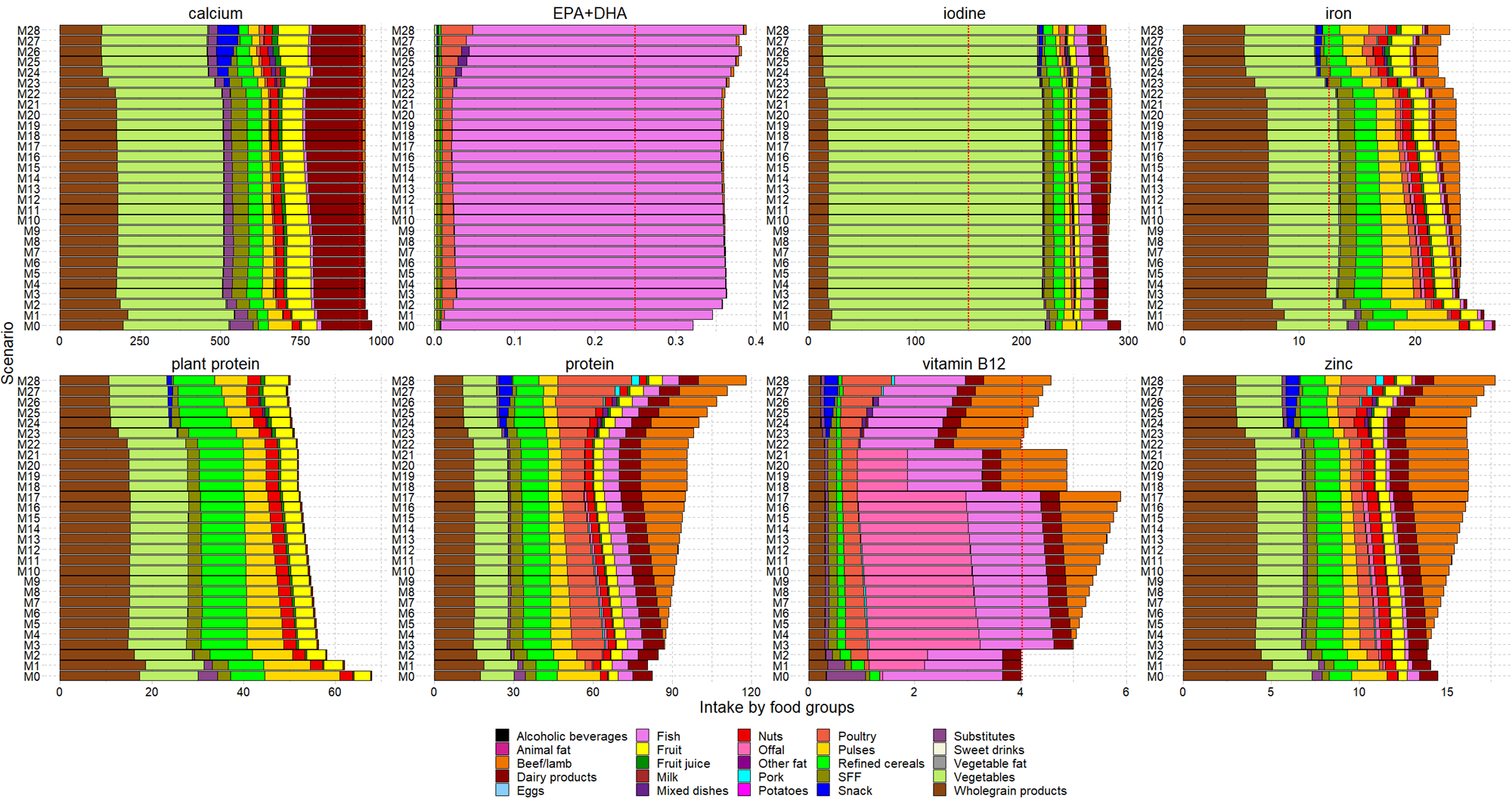


Abbreviations: DHA, docosahexaenoic acid; EI, energy intake; EPA, eicosapentaenoic acid; M:model; SFF, Sweet and fat foods

Units were mg for calcium, iron, and zinc, µg for iodine, and vitamin B12, kcal for energy intake and g for DHA, EPA and protein.

^1^Mixed dishes include sandwich, dishes such as pizza, hamburger, ravioli, panini, salted pancake, sweet and fat foods (SFF) include croissants, pastries, chocolate, biscuits, milky desserts, ice cream, honey and marmalade, cakes, chips, salted oilseeds, salted biscuits, and sweet drinks include fruit nectar, syrup, soda (with or without sugar)

^2^M0 to M28 denote models imposing GHGe of 1.2 to 6.8 kgCO2eq/d by increments of 0.2

1. High Council of Public Health. Statement related to the revision of the 2017-2021 French Nutrition and Health Programme’s dietary guidelines for adults [Internet]. Paris: Haut Conseil de la Santé Publique; 2017 févr [cité 5 févr 2019]. Disponible sur: https://www.hcsp.fr/explore.cgi/avisrapportsdomaine?clefr=653

2. French Agency for Food, Environmental and Occupational Health Safety (Anses). Actualisation des repères du PNNS : élaboration des références nutritionnelles [Internet]. Maison Alfort: ANSES; 2016 déc. Disponible sur: Available from: https://www.anses.fr/fr/system/files/NUT2012SA0103Ra-2.pdf

3. Chaltiel D, Adjibade M, Deschamps V, Touvier M, Hercberg S, Julia C, et al. Programme National Nutrition Santé – guidelines score 2 (PNNS-GS2): development and validation of a diet quality score reflecting the 2017 French dietary guidelines – CORRIGENDUM. Br J Nutr. 14 janv 2021;125(1):118‑20.

4. Seconda L, Baudry J, Allès B, Boizot-Szantai C, Soler LG, Galan P, et al. Comparing nutritional, economic, and environmental performances of diets according to their levels of greenhouse gas emissions. Climatic Change. 13 avr 2018;148.

5. Pointereau P, Langevin B, Gimaret M. ResearchGate. 2012 [cité 16 juin 2017]. DIALECTE, a comprehensive and quick tool to assess the agro-environmental performance of farms (PDF Download Available). Disponible sur: https://www.researchgate.net/publication/265288497_DIALECTE_a_comprehensive_and_quick_tool_to_assess_the_agro-environmental_performance_of_farms

6. Weidema BP, Meeusen MJG. Agricultural Data for Life Cycle Assessments. La Hague, Pays-Bas; 2000. 189 p.

7. Clune S, Crossin E, Verghese K. Systematic review of greenhouse gas emissions for different fresh food categories. Journal of Cleaner Production. 1 janv 2017;140, Part 2:766‑83.

8. ADEME [Internet]. [cité 9 juin 2017]. L’évaluation environnementale en agriculture - L’outil AGRIBALYSE®. Disponible sur: http://www.ademe.fr/expertises/produire-autrement/production-agricole/passer-a-laction/dossier/levaluation-environnementale-agriculture/loutil-agribalyser

9. Frischknecht R, Jungbluth N, Althaus HJ, Bauer C, Doka G, Dones R, et al. Implementation of Life Cycle Impact Assessment Methods Report 3.

10. ADEME. Dia’terre®. Synthèse du guide de la méthode et guide des valeurs. 2015.

11. de Ponti T, Rijk B, van Ittersum MK. The crop yield gap between organic and conventional agriculture. Agricultural Systems. avr 2012;108:1‑9.

12. Ministère de l’agriculture et de l’alimentation - agreste - La statistique, l’évaluation et la prospective agricole [Internet]. [cité 9 juin 2017]. Disponible sur: http://www.agreste.agriculture.gouv.fr/

13. Colomb V, A. Colsaet, S. Ait-Amar, C. Basset-Mens, G. Mevel, V. To, et al. AGRIBALYSE: the French public LCI database for agricultural products [Internet]. Unpublished; 2015 [cité 28 sept 2017]. Disponible sur: http://rgdoi.net/10.13140/RG.2.1.2586.0240

14. Hallberg L, Hulthén L. Prediction of dietary iron absorption: an algorithm for calculating absorption and bioavailability of dietary iron. Am J Clin Nutr. mai 2000;71(5):1147‑60.

15. Hallberg L, Brune M, Rossander L. Iron absorption in man: ascorbic acid and dose-dependent inhibition by phytate. Am J Clin Nutr. janv 1989;49(1):140‑4.

16. Armah SM, Carriquiry A, Sullivan D, Cook JD, Reddy MB. A complete diet-based algorithm for predicting nonheme iron absorption in adults. J Nutr. juill 2013;143(7):1136‑40.

17. Miller LV, Krebs NF, Hambidge KM. A mathematical model of zinc absorption in humans as a function of dietary zinc and phytate. J Nutr. janv 2007;137(1):135‑41.

18. GBD 2019 Risk Factors Collaborators. Global burden of 87 risk factors in 204 countries and territories, 1990-2019: a systematic analysis for the Global Burden of Disease Study 2019. Lancet. 17 oct 2020;396(10258):1223‑49.

19. Fouillet H, Dussiot A, Perraud E, Wang J, Huneau JF, Kesse-Guyot E, et al. Plant to animal protein ratio in the diet: nutrient adequacy, long-term health and environmental pressure [Internet]. medRxiv; 2022 [cité 19 févr 2023]. p. 2022.05.20.22275349. Disponible sur: https://www.medrxiv.org/content/10.1101/2022.05.20.22275349v1
